# Inhibiting SCAP/SREBP exacerbates liver injury and carcinogenesis in murine nonalcoholic steatohepatitis

**DOI:** 10.1172/JCI151895

**Published:** 2022-06-01

**Authors:** Satoshi Kawamura, Yuki Matsushita, Shigeyuki Kurosaki, Mizuki Tange, Naoto Fujiwara, Yuki Hayata, Yoku Hayakawa, Nobumi Suzuki, Masahiro Hata, Mayo Tsuboi, Takahiro Kishikawa, Hiroto Kinoshita, Takuma Nakatsuka, Masaya Sato, Yotaro Kudo, Yujin Hoshida, Atsushi Umemura, Akiko Eguchi, Tsuneo Ikenoue, Yoshihiro Hirata, Motonari Uesugi, Ryosuke Tateishi, Keisuke Tateishi, Mitsuhiro Fujishiro, Kazuhiko Koike, Hayato Nakagawa

**Affiliations:** 1 Department of Gastroenterology, The University of Tokyo, Tokyo, Japan.; 2 Department of Internal Medicine, University of Texas Southwestern Medical Center, Dallas, Texas, USA.; 3 Division of Gastroenterology, Institute for Adult Diseases, Asahi Life Foundation, Tokyo, Japan.; 4 Department of Gastroenterology and Hepatology, Kyoto Prefectural University of Medicine, Kyoto, Japan.; 5 Department of Gastroenterology and Hepatology, Mie University, Mie, Japan.; 6 Division of Clinical Genome Research and; 7 Division of Advanced Genome Medicine, The Institute of Medical Science, The University of Tokyo, Tokyo, Japan.; 8 Institute for Chemical Research and Institute for Integrated Cell-Material Sciences (WPI-iCeMS), Kyoto University, Kyoto, Japan.

**Keywords:** Gastroenterology, Hepatitis

## Abstract

Enhanced de novo lipogenesis mediated by sterol regulatory element–binding proteins (SREBPs) is thought to be involved in nonalcoholic steatohepatitis (NASH) pathogenesis. In this study, we assessed the impact of SREBP inhibition on NASH and liver cancer development in murine models. Unexpectedly, SREBP inhibition via deletion of the SREBP cleavage–activating protein (SCAP) in the liver exacerbated liver injury, fibrosis, and carcinogenesis despite markedly reduced hepatic steatosis. These phenotypes were ameliorated by restoring SREBP function. Transcriptome and lipidome analyses revealed that SCAP/SREBP pathway inhibition altered the fatty acid (FA) composition of phosphatidylcholines due to both impaired FA synthesis and disorganized FA incorporation into phosphatidylcholine via lysophosphatidylcholine acyltransferase 3 (LPCAT3) downregulation, which led to endoplasmic reticulum (ER) stress and hepatocyte injury. Supplementation with phosphatidylcholines significantly improved liver injury and ER stress induced by SCAP deletion. The activity of the SCAP/SREBP/LPCAT3 axis was found to be inversely associated with liver fibrosis severity in human NASH. SREBP inhibition also cooperated with impaired autophagy to trigger liver injury. Thus, excessively strong and broad lipogenesis inhibition was counterproductive for NASH therapy; this will have important clinical implications in NASH treatment.

## Graphical abstract

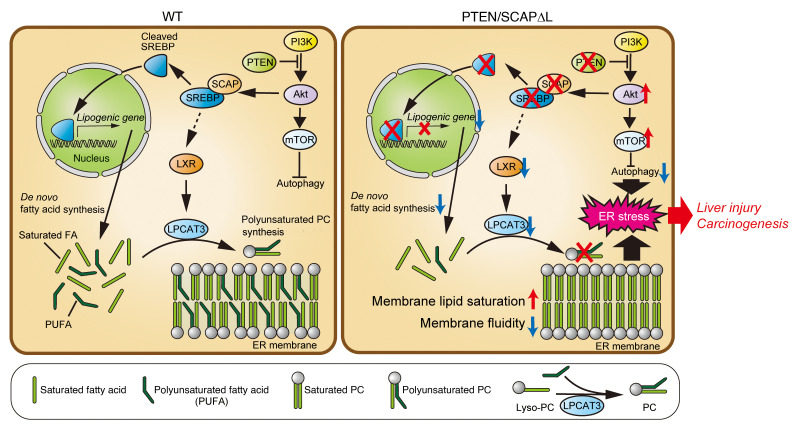

## Introduction

Nonalcoholic steatohepatitis (NASH) is a severe form of nonalcoholic fatty liver disease (NAFLD) that is characterized by hepatic steatosis, inflammation, hepatocellular injury, and fibrosis, which lead to progression of cirrhosis and hepatocellular carcinoma (HCC) (1). Although lipid influx to the liver from insulin-resistant adipose tissue and dietary fat is increased in patients with NASH, hepatic de novo lipogenesis is paradoxically enhanced (2, 3). In healthy subjects, 80% of hepatic fatty acids (FAs) are derived from free FA influx from adipose tissue, and those from de novo lipogenesis account for only 5%, whereas in patients with NAFLD, the proportion from de novo lipogenesis increases to 26%. This lipid metabolism disturbance is considered to cause lipotoxicity-induced hepatocyte death, leading to subsequent fibrosis and HCC (4–6). Based on these concepts, several drugs targeting enzymes involved in hepatic de novo lipogenesis, such as acetyl-CoA carboxylase (ACC), FA synthase (FASN), and stearoyl-CoA desaturase 1 (SCD1), are under development (7, 8).

Sterol regulatory element–binding proteins (SREBPs) are a family of transcription factors recognized as the master regulators of lipogenesis, consisting of 3 isoforms, SREBP-1a, SREBP-1c, and SREBP-2. SREBP-1c mainly promotes the transcription of genes involved in FA and triacylglycerol synthesis, whereas SREBP-2 mainly regulates cholesterol synthesis (9). SREBP-1a can stimulate both FA and cholesterol synthesis; however, these pathways overlap significantly and have complex interactions (10, 11). SREBPs exist as membrane-bound precursors at the endoplasmic reticulum (ER) and are transported to the Golgi apparatus by the escort protein SREBP cleavage–activating protein (SCAP) to activate lipid biosynthesis, which is a critical process for SREBP activation. Subsequently, SREBPs are proteolytically cleaved to release their NH_2_ terminal domain as an active form that can enter the nucleus to stimulate the transcription of target genes (12). Importantly, the lipogenic enzymes ACC, FASN, and SCD1 are all regulated by SREBP, and therefore SREBP inhibition could be an effective therapeutic strategy against NAFLD. Because SREBP-mediated de novo lipogenesis is often also upregulated in HCC, SREBP has attracted attention as a promising therapeutic target against HCC (13). However, to the best of our knowledge, there is no conclusive experimental evidence demonstrating that SREBP inhibition can prevent NASH or NASH-related HCC development.

Therefore, in this study, we investigated the impact of SREBP inhibition on NASH and HCC development. We used a well-known NASH-HCC mouse model: a liver-specific phosphatase and tensin homolog (PTEN) knockout mouse (PTEN^ΔL^) generated by crossing a *Pten^fl/fl^* mouse and an albumin-Cre mouse (*Alb-Cre*). PTEN^ΔL^ mice show constitutive upregulation of SREBP due to PI3K/Akt pathway activation, which leads to spontaneous fatty liver and subsequent HCC development (14, 15). To inhibit the SREBP pathway in PTEN^ΔL^ mice, we additionally ablated the *SCAP* gene in the liver because the single-knockout SREBP isoform reportedly induces compensation of other isoforms, whereas *SCAP* knockout almost completely inhibits SREBP activation (16–18). The results confirmed that the SREBP pathway is required for hepatic steatosis in PTEN^ΔL^ mice; however, unexpectedly, SCAP/SREBP pathway inhibition markedly exacerbated liver injury, fibrosis, and carcinogenesis in PTEN^ΔL^ mice. Accordingly, we investigated the underlying mechanisms of this phenotype.

## Results

### Liver-specific PTEN/SCAP double-knockout mice exhibit spontaneous severe liver injury.

**Figure 1:**
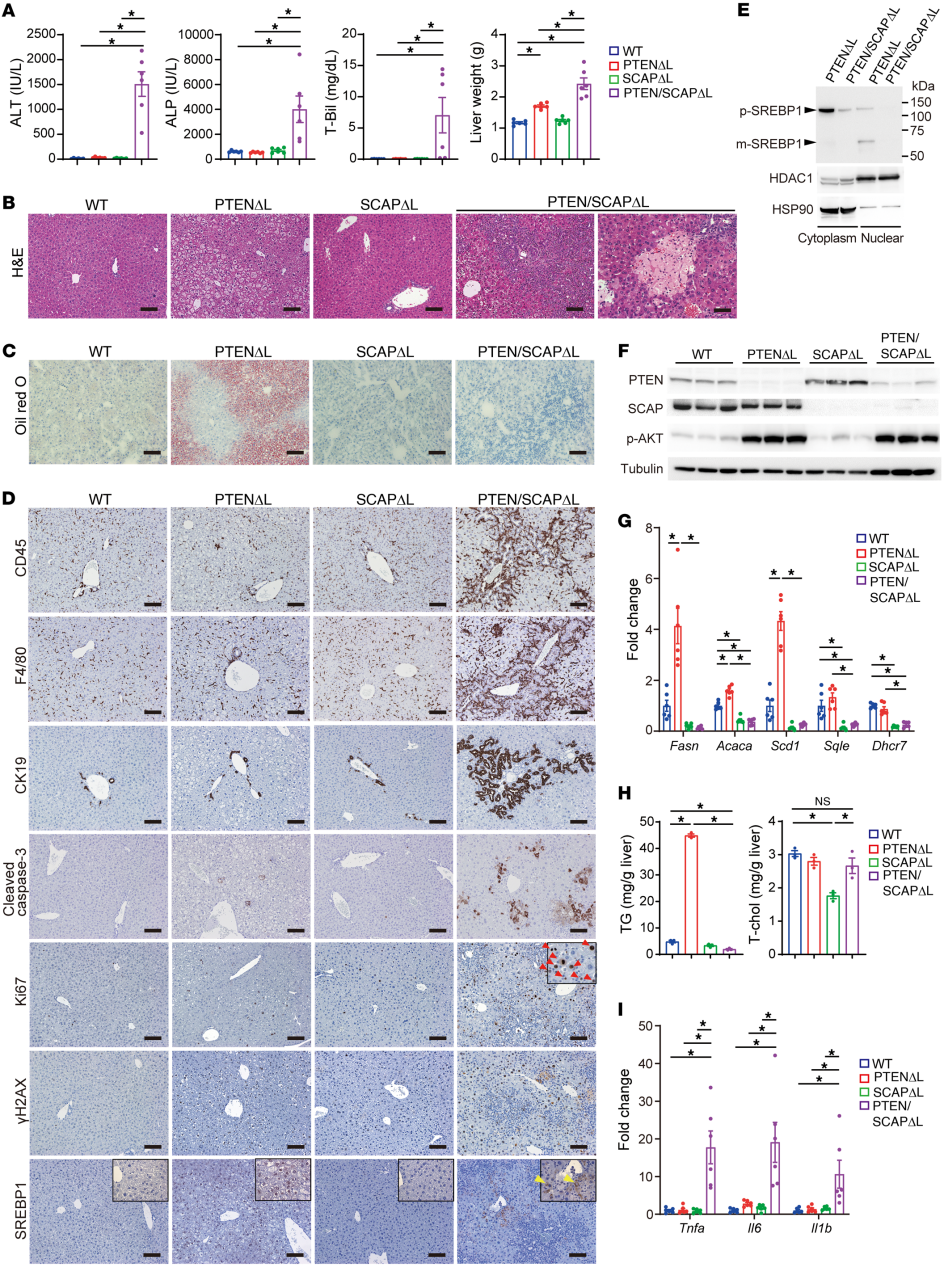
Liver-specific PTEN/SCAP double-knockout mice exhibit severe liver injury. (**A**) Serum levels of ALT, ALP, total bilirubin, and liver weight in 5-week-old WT, PTEN^ΔL^, SCAP^ΔL^, and PTEN/SCAP^ΔL^ mice (*n* = 6 per group). (**B** and **C**) H&E (**B**) and oil red O (**C**) staining images of livers from mice indicated in **A**. Right H&E image shows the focal necrosis of liver parenchyma in PTEN/SCAP^ΔL^ mice. Scale bars: 50 μm (H&E, furthest right); 100 μm (all others). (**D**) IHC images of indicated proteins in livers from mice indicated in **A**. Scale bars: 100 μm. Red arrowheads, Ki67-expressing hepatocytes; yellow arrowheads, hepatocytes expressing SREBP-1 in the cytoplasm but not the nucleus. Enlarged high magnification images of Ki67 and SREBP-1 staining are shown in Supplemental Figure 1B. (**E**) Cytoplasmic and nuclear protein fractions were extracted from 5-week-old PTEN^ΔL^ and PTEN/SCAP^ΔL^ mouse livers, and the cytoplasmic protein levels of precursor SREBP-1 (p-SREBP1) and nuclear mature form of SREBP-1 (m-SREBP1) were analyzed by WB. (**F**) WB analyses of indicated proteins in livers from mice indicated in **A**. (**G**) Relative expression levels of lipogenic genes determined by real-time PCR in livers from mice indicated in **A** (*n* = 6 per group). *P < 0.05. (**H**) Hepatic TG and total cholesterol content of livers from mice indicated in **A** (*n* = 3 per group). (**I**) Relative expression levels of inflammatory cytokines determined by real-time PCR in livers from mice indicated in **A** (*n* = 6 per group). *P < 0.05. All statistical data were assessed using 1-way ANOVA with Tukey’s multiple comparison test (**A**, **G**, **H**, and **I**) and are presented as mean ± SEM.

To examine the effects of SCAP/SREBP pathway inhibition in PTEN^ΔL^ mice, we generated liver-specific PTEN/SCAP double-knockout mice by crossing PTEN^ΔL^ mice with *Scap^fl/fl^* mice (PTEN/SCAP^ΔL^). PTEN/SCAP^ΔL^ mice were born at the expected frequency and appeared normal. However, PTEN/SCAP^ΔL^ mice exhibited abdominal distention at around 5 weeks of age, sometimes accompanied by jaundice. Therefore, we first compared the phenotypes of 5-week-old PTEN/SCAP^ΔL^ mice to those of WT Cre-negative control *Pten^fl/fl^/Scap^fl/fl^* mice, PTEN^ΔL^, and liver-specific SCAP single-knockout mice (SCAP^ΔL^) that were generated by crossing *Scap^fl/fl^* and *Alb-Cre* mice. In PTEN/SCAP^ΔL^ mice, serum levels of alanine aminotransferase (ALT), alkaline phosphatase (ALP), and total bilirubin were markedly elevated, and liver weight was significantly increased (Figure 1A). There were no differences in the degree of liver damage between male and female mice (data not shown). Histologically, PTEN^ΔL^ mice showed mild hepatic steatosis at this age, while hepatic steatosis was almost abolished by additional SCAP deletion, as confirmed by oil red O staining (Figure 1, B and C). However, PTEN/SCAP^ΔL^ mice showed marked periportal inflammation and focal necrosis of the liver parenchyma (Figure 1B). Single knockout of SCAP did not induce apparent liver injury or elevated liver enzyme levels in serum (Figure 1, A and B).

IHC analyses indicated marked infiltration of inflammatory cells into the periportal area, accompanied by increased CK19-expressing ductal cells (i.e., ductular reaction) in PTEN/SCAP^ΔL^ mice (Figure 1D). iNOS and arginase-1 were stained to determine the phenotype of infiltrating macrophages, i.e., M1 or M2. iNOS^+^ M1-like macrophages increased predominantly in PTEN/SCAP^ΔL^ mice (Supplemental Figure 1A; supplemental material available online with this article; https://doi.org/10.1172/JCI151895DS1). The frequency of cleaved caspase-3–positive cells was significantly higher in the liver of PTEN/SCAP^ΔL^ mice than in those of other mouse groups; as a result, the number of Ki67-positive proliferating hepatocytes was also increased (Figure 1D). DNA damage marker γH2AX-positive hepatocytes were also frequently observed in PTEN/SCAP^ΔL^ mice. Nuclear expression of SREBP-1 was significantly increased in the hepatocytes of PTEN^ΔL^ mice, but abolished in PTEN/SCAP^ΔL^ mice (Figure 1D). Although some hepatocytes in PTEN/SCAP^ΔL^ mice showed cytoplasmic staining of SREBP-1, the nuclei of these cells were not stained, indicating that SREBP activation was inhibited in PTEN/SCAP^ΔL^ mice (Supplemental Figure 1B). Inhibition of nuclear translocation of SREBP-1 in PTEN/SCAP^ΔL^ mice was confirmed by Western blotting (WB) of nuclear extracts (Figure 1E). WB analyses showed the efficient deletion of PTEN and/or SCAP protein in the liver, in accordance with the respective genotypes (Figure 1F). The deletion of *PTEN* significantly increased Akt phosphorylation, which was not affected by additional *SCAP* deletion (Figure 1F). Hepatic mRNA levels of the SREBP-1 target genes involved in de novo lipogenesis, *Fasn*, *Acaca*, and *Scd1*, were significantly increased in PTEN^ΔL^ mice, but significantly decreased in SCAP^ΔL^ and PTEN/SCAP^ΔL^ mice (Figure 1G). *Sqle* and *Dhcr7*, which are SREBP-2 target genes involved in cholesterol biosynthesis, were also significantly decreased in SCAP^ΔL^ and PTEN/SCAP^ΔL^ mice, whereas their expression levels in PTEN^ΔL^ mice were comparable to those in WT mice. These findings indicate that SREBP function was efficiently inhibited in PTEN/SCAP^ΔL^ mouse livers. Consistent with lipogenic gene expression data, hepatic triglyceride (TG) content was increased in PTEN^ΔL^ mice, but significantly decreased in SCAP^ΔL^ and PTEN/SCAP^ΔL^ mice (Figure 1H). Hepatic cholesterol content was also decreased in SCAP^ΔL^ mice, whereas hepatic cholesterol levels in PTEN/SCAP^ΔL^ and WT mice were comparable, probably due to decreased biliary cholesterol secretion caused by severe liver injury. Consistent with histological inflammation, expression levels of the proinflammatory cytokines *Tnfa*, *Il6*, and *Il1b*, which are reportedly involved in NASH and NASH-related hepatocarcinogenesis, were significantly increased in PTEN/SCAP^ΔL^ mice (Figure 1I and refs. 6, 19, 20). Caspase-2 is an alternative pathway to activating SREBP in NASH (21). Although the amounts of precursor and cleaved caspase-2 increased in PTEN/SCAP^ΔL^ mice (Supplemental Figure 2), activation of the SREBP pathway was almost completely inhibited (Figure 1, D, E, and G), suggesting that caspase-2 does not play a major role in our experimental model. Together, these results indicate that SCAP deletion inhibited hepatic lipogenesis and steatosis in PTEN^ΔL^ mice, but unexpectedly induced severe liver injury and inflammation.

### Deletion of SCAP in PTEN^ΔL^ mice induces severe liver fibrosis and accelerates liver cancer development.

**Figure 2:**
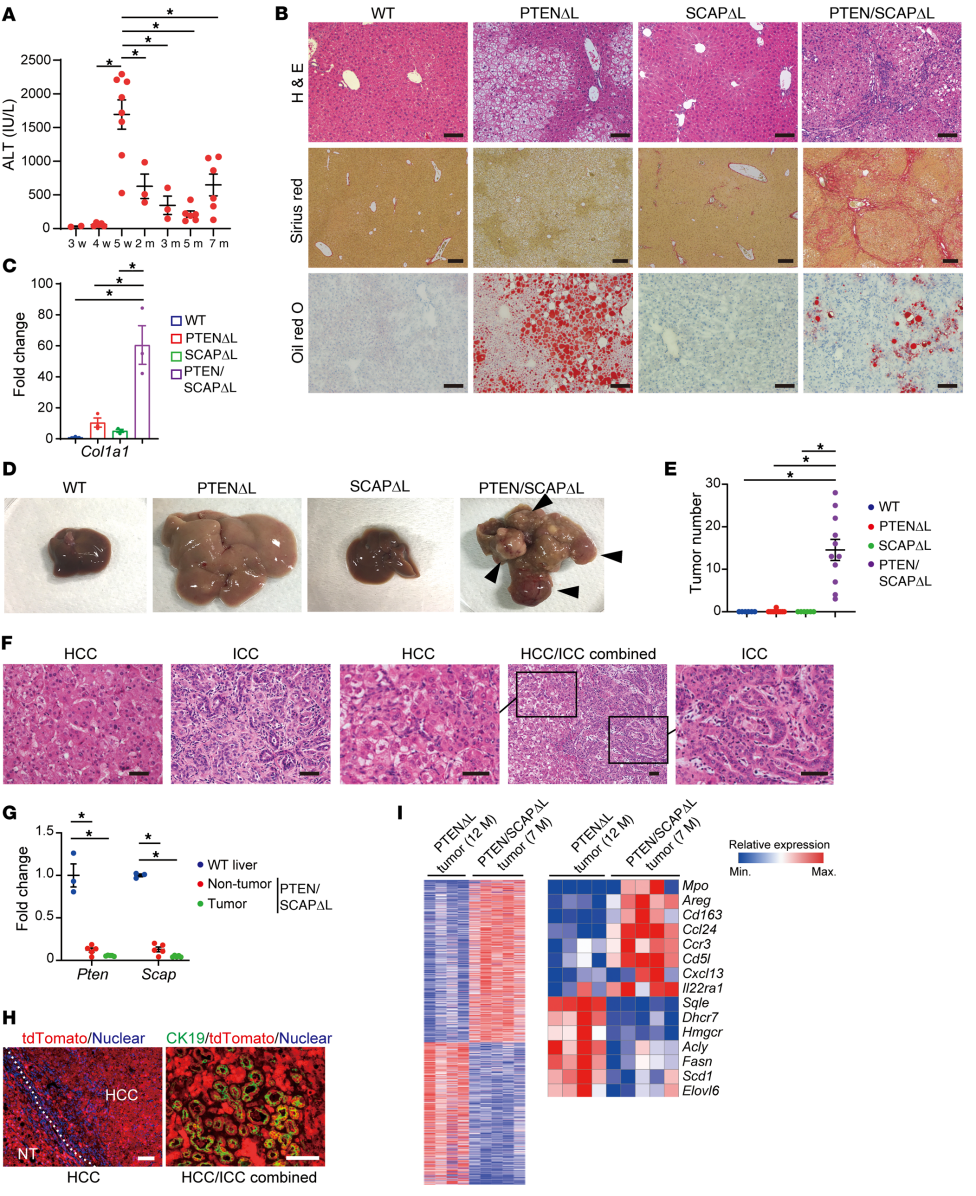
Deletion of SCAP in PTEN^ΔL^ mice induces severe liver fibrosis and accelerates liver cancer development. (**A**) Time course of serum ALT in PTEN/SCAP^ΔL^ mice (sample sizes by age: 3 weeks, *n* = 2; 4 weeks, *n* = 5; 5 weeks, *n* = 8; 2 months, *n* = 3; 3 months, *n* = 3; 5 months, *n* = 6; 7 months, *n* = 6). (**B**) H&E, Sirius red, and oil red O staining images of livers from 5-month-old WT, PTEN^ΔL^, SCAP^ΔL^, and PTEN/SCAP^ΔL^ mice. Scale bars: 250 μm (Sirius red); 100 μm (all others). (**C**) Relative expression levels of *Col1a1* mRNA determined by real-time PCR in livers from mice indicated in **B** (*n* = 3 per group). (**D** and **E**) Representative liver images (**D**) and tumor numbers (**E**) for 7-month-old mice of each genotype (WT and SCAP^ΔL^, *n* = 6; PTEN^ΔL^ and PTEN/SCAP^ΔL^, *n* = 11). Arrowheads, liver tumors. (**F**) H&E images of liver tumors from PTEN/SCAP^ΔL^ mice. Scale bars: 50 μm. (**G**) Relative expression levels of *Pten* and *Scap* mRNAs by real-time PCR in liver tissues of 7-month-old WT mice and nontumor liver tissues and tumor tissues of 7-month-old PTEN/SCAP^ΔL^ mice (WT, *n* = 3; nontumor and tumor tissues of PTEN/SCAP^ΔL^ mice, *n* = 5). (**H**) Immunofluorescence staining of tdTomato in HCC (left panel) and double immunofluorescence staining of tdTomato and ductal cell marker CK19 in HCC/ICC combined tumor (right panel) derived from 7-month-old PTEN/SCAP^ΔL^;tdTomato mice. Scale bars: 100 μm. (**I**) Heatmaps show significant differentially expressed genes between PTEN^ΔL^ tumors and PTEN/SCAP^ΔL^ tumors (PTEN^ΔL^, *n* = 4; PTEN/SCAP^ΔL^, *n* = 5). Left panel, total differentially expressed genes. Right panel, selected differentially expressed genes. All statistical data were assessed using 1-way ANOVA with Tukey’s multiple comparison test (**A**, **C**, **E**, and **G**). Data are presented as mean ± SEM. *P < 0.05.

Although 5% of PTEN/SCAP^ΔL^ mice died at 5 to 6 weeks of age, all remaining mice, including those that exhibited jaundice, survived for longer than 6 months. However, all PTEN/SCAP^ΔL^ mice became sick; this was accompanied by marked abdominal distention at around 7 months of age. Therefore, we analyzed the time course of liver phenotypes in PTEN/SCAP^ΔL^ mice from 3 weeks to 7 months of age. Serum levels of ALT were within normal range until 4 weeks, but increased dramatically at 5 weeks of age (Figure 2A). These levels gradually decreased, whereas high levels of ALT (~300–500 IU/L) were sustained. Serum levels of total bilirubin also declined at 2 months of age (Supplemental Figure 3A). Liver histology appeared almost normal until 4 weeks, but exhibited severe liver injury at 5 weeks (Supplemental Figure 3B). Consistent with the changes in ALT and bilirubin levels, periportal inflammation and hepatocyte necrosis were gradually attenuated over time (Supplemental Figure 3B). At 5 months, although marked hepatic steatosis was observed in PTEN^ΔL^ mice, PTEN/SCAP^ΔL^ mice showed only mild lipid accumulation (Figure 2B). Notably, as a consequence of chronic liver damage, 5-month-old PTEN/SCAP^ΔL^ mice exhibited severe liver fibrosis, nearly cirrhosis, accompanied by a significant increase in type 1 collagen α1 (*Col1a1*) mRNA (Figure 2, B and C), whereas PTEN^ΔL^ and SCAP^ΔL^ mice showed only very mild fibrosis at the same age.

At 7 months of age, all male PTEN/SCAP^ΔL^ mice developed multiple liver tumors, whereas only a single very small tumor was observed in 1 PTEN^ΔL^ mouse despite all PTEN^ΔL^ mice having enlarged whitish livers due to severe fatty change (Figure 2, D and E). No liver tumors were found in WT or SCAP^ΔL^ mice. Histologically, although more than 90% of tumors arising in PTEN/SCAP^ΔL^ mice were HCC, intrahepatic cholangiocarcinoma (ICC) and combined HCC/ICC tumors were also observed (Figure 2F). To rule out the possibility that these tumors were derived from cells escaping Cre-mediated recombination, we analyzed *Scap* and *Pten* mRNA levels in tumor tissues. Both *Scap* and *Pten* mRNAs were significantly lower in tumor tissues and nontumor tissues of 7-month-old PTEN/SCAP^ΔL^ mice than in liver tissues of WT mice of the same age (Figure 2G). To further confirm this finding, we crossed PTEN/SCAP^ΔL^ mice with *Rosa26*-*Lox*-*Stop*-*Lox*-*tdTomato* reporter mice (PTEN/SCAP^ΔL^;tdTomato) to label the recombined cells. All tumors, including cholangiocarcinoma lesions arising in PTEN/SCAP^ΔL^;tdTomato mice, expressed tdTomato (Figure 2H), indicating that these tumors had originated from cells with genetic recombination.

To explore whether a specific genetic alteration promotes tumorigenesis, we conducted whole-exome sequencing of tumor samples obtained from 7-month-old PTEN/SCAP^ΔL^ mice (*n* = 8). We also analyzed liver tumor samples obtained from 12-month-old PTEN^ΔL^ mice for comparison (*n* = 7). As previously reported (14), 12-month-old PTEN^ΔL^ mice developed multiple liver tumors (Supplemental Figure 4A). Interestingly, we identified several commonly mutated genes in tumors derived from PTEN^ΔL^ mice, including *Adam11*, *Dhx9*, *Spata31d1c* (87.5%), and *Nudt9* (71.4%), whereas tumors from PTEN/SCAP^ΔL^ mice had more heterogeneous mutational profiles (Supplemental Figure 4, B and C). We also analyzed the mutational signatures of these tumors, which can reflect the mutational process (22). PTEN/SCAP^ΔL^ tumors had specific mutational signatures (8, 11, 12, 17, and 25, according to Catalogue of Somatic Mutations in Cancer [COSMIC] nomenclature) that were not observed in PTEN^ΔL^ tumors (Supplemental Figure 4D). It is especially noteworthy that signature 12 is reportedly exclusive to liver cancer and strongly enriched in patients with chronic viral hepatitis (23). Furthermore, we conducted RNA-Seq analyses using the same tumor samples, and a total of 1529 significant differentially expressed genes were identified (Figure 2I). Lipogenic genes, particularly those involved in cholesterol metabolism, were significantly downregulated in PTEN/SCAP^ΔL^ tumors compared with PTEN^ΔL^ tumors, while inflammatory pathways involved in hepatocarcinogenesis, such as the CCL24/CCR3 axis, were significantly upregulated (ref. 24 and Figure 2I). These findings suggest that PTEN^ΔL^ and PTEN/SCAP^ΔL^ mice have distinct carcinogenesis processes and that chronic liver injury and inflammation play key roles in enhanced carcinogenesis in PTEN/SCAP^ΔL^ mice. On the other hand, carcinogenesis in PTEN/SCAP^ΔL^ mice was less dependent on lipogenesis.

We also analyzed the effects of SCAP deletion in another NASH-HCC mouse model, the choline-deficient, l-amino acid–defined, high-fat-diet (CDAHFD) model (25). *Scap^fl/fl^* and SCAP^ΔL^ mice were placed on CDAHFD for 32 weeks, starting at 6 weeks of age, and tumorigenicity was assessed. SCAP^ΔL^ mice exhibited significantly increased liver tumor development accompanied by significant elevation of IL-6, despite a mild reduction in hepatic steatosis (Supplemental Figure 5, A–D). Furthermore, we analyzed cell death by TUNEL staining. As reported previously (26), apoptotic cells and necrotic cells were stained as nuclear fragmentation and diffuse cytoplasmic patterns, respectively, in the CDAHFD model. TUNEL^+^ dying cells were more frequently observed in SCAP^ΔL^ mice compared with *Scap^fl/fl^* mice (Supplemental Figure 5E). Therefore, the enhanced liver injury and carcinogenesis caused by SCAP deletion is not a phenomenon specific to the PTEN^ΔL^ mouse model.

### Restoration of SREBP function in PTEN/SCAP^ΔL^ mice ameliorates liver injury, fibrosis, and tumorigenesis.

**Figure 3:**
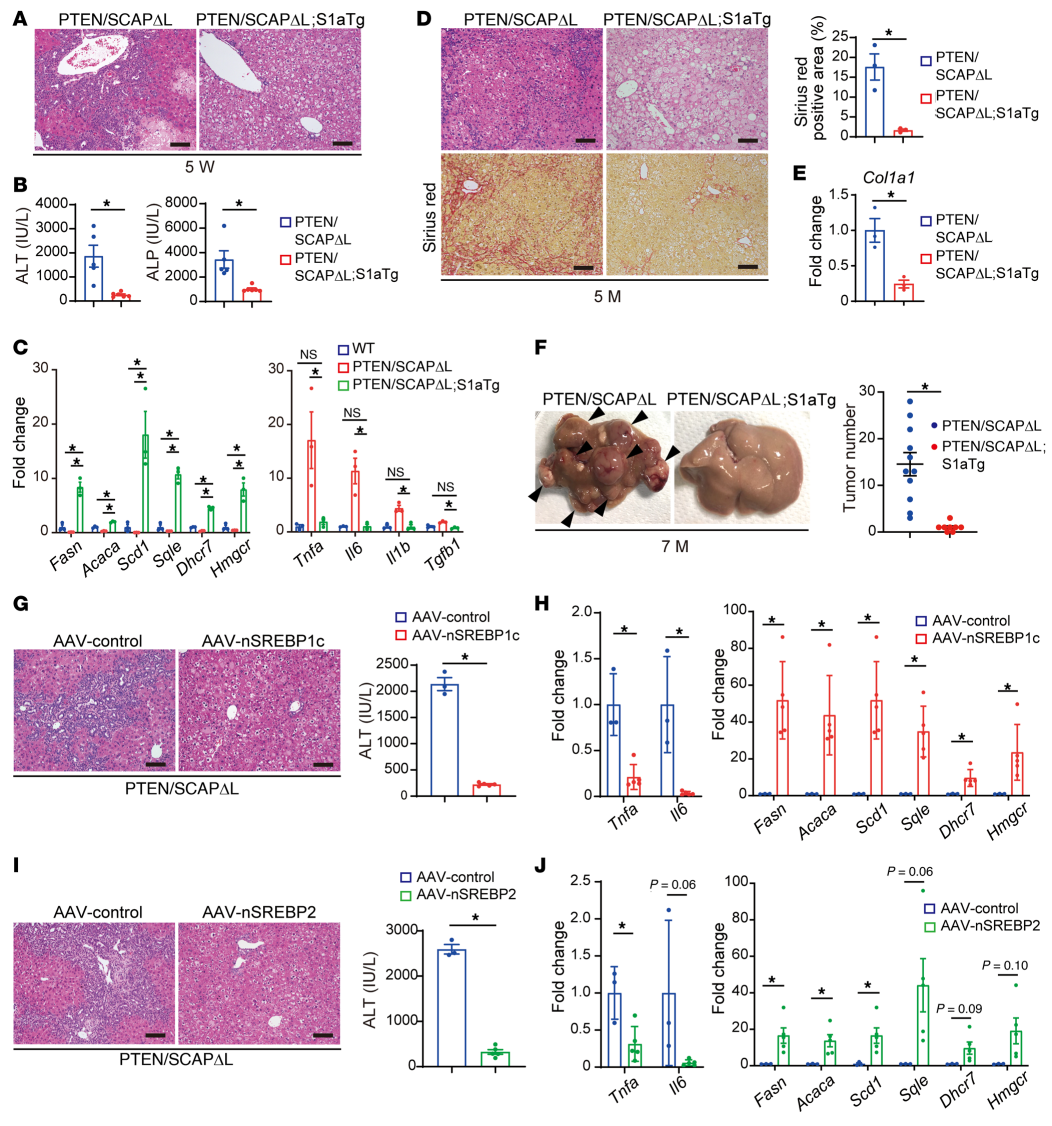
Restoration of SREBP function in PTEN/SCAP^ΔL^ mice ameliorates liver injury, fibrosis, and carcinogenesis. (**A** and **B**) H&E images of livers (**A**) and serum levels of ALT and ALP (**B**) for 5-week-old PTEN/SCAP^ΔL^ and PTEN/SCAP^ΔL^;S1aTg mice. Scale bars: 100 μm. (PTEN/SCAP^ΔL^, *n* = 5; PTEN/SCAP^ΔL^;S1aTg, *n* = 6). (**C**) Relative expression levels of lipogenic genes determined by real-time PCR in livers of 5-week-old WT, PTEN/SCAP^ΔL^, and PTEN/SCAP^ΔL^;S1aTg mice (*n* = 3 per group). (**D**) H&E and Sirius red images of livers from 5-month-old PTEN/SCAP^ΔL^ and PTEN/SCAP^ΔL^;S1aTg mice. Scale bars: 100 μm. Bar graph shows Sirius red–positive area (*n* = 3 per group). (**E**) Relative expression levels of *Col1a1* mRNA determined by real-time PCR in livers from 5-month-old PTEN/SCAP^ΔL^ and PTEN/SCAP^ΔL^;S1aTg mice (*n* = 3 per group). (**F**) Representative liver images and tumor numbers for 7-month-old PTEN/SCAP^ΔL^ and PTEN/SCAP^ΔL^;S1aTg mice (PTEN/SCAP^ΔL^, *n* = 11; PTEN/SCAP^ΔL^;S1aTg, *n* = 8). Arrowheads indicate liver tumors. (**G**–**J**) We intravenously injected 4-week-old PTEN/SCAP^ΔL^ mice with 1.5 × 10^11^ genome copies of AAV-control, AAV-nSREBP-1c, or AAV-nSREBP-2 and analyzed 1 week after injection (AAV-control, *n* = 3 for each experiment; AAV-nSREBP-1c and AAV-nSREBP-2, *n* = 5). (**G** and **I**) H&E images of livers and serum ALT. Scale bars: 100 μm. (**H** and **J**) Relative expression levels of inflammatory cytokines and lipogenic genes determined by real-time PCR. Statistical data in **B**, **D**, and **E**–**J** were assessed using Student’s *t* test and in **C** using 1-way ANOVA with Tukey’s multiple comparison test. Data are presented as mean ± SEM. *P < 0.05.

Although SCAP plays a critical role in SREBP activation, it may have other functions whose inhibition could result in liver injury in PTEN^ΔL^ mice. To determine whether impaired SREBP activation is a causal factor for liver injury in PTEN/SCAP^ΔL^ mice, we crossed PTEN/SCAP^ΔL^ mice with transgenic mice expressing a truncated active form of SREBP-1a (PTEN/SCAP^ΔL^;S1aTg) driven by the phosphoenolpyruvate carboxykinase (PEPCK) promoter, which produces high expression levels in the liver (27). Although SREBP-1c is the predominant SREBP-1 isoform in the liver, we first introduced SREBP-1a into PTEN/SCAP^ΔL^ mice using a transgenic approach because it would reportedly stimulate both FA and cholesterol synthesis, thereby enabling complete restoration of SREBP function (9). Strikingly, liver injury and inflammation were dramatically improved in 5-week-old PTEN/SCAP^ΔL^;S1aTg mice compared with PTEN/SCAP^ΔL^ mice, and PTEN/SCAP^ΔL^;S1aTg mouse liver histology revealed only mild fatty change, that is, so-called “simple steatosis” (Figure 3A). Serum ALT and ALP levels were markedly decreased in PTEN/SCAP^ΔL^;S1aTg mice (Figure 3B), and no PTEN/SCAP^ΔL^;S1aTg mice exhibited apparent jaundice at this time point. We confirmed that the expression levels of enzymes involved in FA and cholesterol metabolism were significantly increased by the introduction of SREBP-1a (Figure 3C). The expression levels of proinflammatory cytokines and profibrotic cytokine *Tgfb1* were markedly decreased in PTEN/SCAP^ΔL^;S1aTg mice compared with PTEN/SCAP^ΔL^ mice, and these were comparable to those of WT mice. Longer-term observation revealed that liver fibrosis was significantly attenuated in PTEN/SCAP^ΔL^;S1aTg mice compared with PTEN/SCAP^ΔL^ mice at 5 months of age and that *Col1a1* mRNA expression was also significantly decreased (Figure 3, D and E). At 7 months of age, liver tumor development was markedly suppressed in PTEN/SCAP^ΔL^;S1aTg mice (Figure 3F). These findings indicate that the exacerbation of liver injury, fibrosis, and tumorigenesis by SCAP deletion in PTEN^ΔL^ mice was caused by impaired SREBP activation.

SREBP-1c and SREBP-2 are considered to preferentially regulate FA metabolism and cholesterol metabolism in the liver, respectively. Therefore, we determined whether SREBP-1c or SREBP-2 dysfunction is responsible for liver injury in PTEN/SCAP^ΔL^ mice. We introduced an HA-tagged truncated active form of SREBP-1c (nSREBP-1c) or SREBP-2 (nSREBP-2) into PTEN/SCAP^ΔL^ mouse livers using adeno-associated virus serotype 8 vectors using the hepatocyte-specific thyroxine-binding globulin promoter (AAV8-TBG) (Supplemental Figure 6A). We also administered AAV8-TBG-EGFP to PTEN/SCAP^ΔL^ mice as a control group (AAV-control) and confirmed that EGFP was introduced into hepatocytes but not into biliary epithelial cells (Supplemental Figure 6B). Both nSREBP-1c and nSREBP-2 introduction significantly improved histological liver injury and inflammation and also suppressed elevation of serum levels of ALT and hepatic inflammatory cytokines in 5-week-old PTEN/SCAP^ΔL^ mice (Figure 3, G–J). However, the introduction of either nSREBP-1c or nSREBP-2 significantly upregulated both genes involved in FA and cholesterol biosynthesis (Figure 3, H and J). Therefore, we could not assess the roles of SREBP-1c and SREBP-2 separately using this method. However, through these experiments, we further confirmed the importance of the SREBP pathway in liver injury of PTEN/SCAP^ΔL^ mice and demonstrated that SREBP pathway inhibition in hepatocytes was the causal factor of this phenotype.

### ER stress is involved in liver injury in PTEN/SCAP^ΔL^ mice.

**Figure 4:**
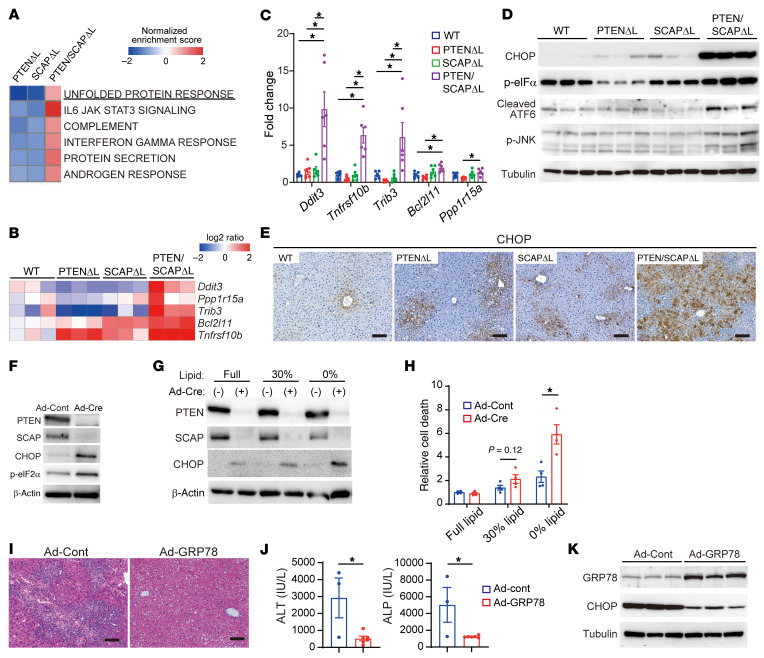
ER stress is involved in liver injury in PTEN/SCAP^ΔL^ mice. (**A**) Pathway analysis of RNA-Seq data. Pathways upregulated in 5-week-old PTEN/SCAP^ΔL^ mouse livers are shown. (**B**) Relative expression levels of ER stress–responsive genes involved in cell death determined by RNA-Seq. Data are expressed as log_2_ ratio compared with WT mice. (**C**) Relative expression levels of genes indicated in **B** were analyzed using real-time PCR (*n* = 6 per group). (**D** and **E**) WB analyses of ER stress markers (**D**) and IHC images of CHOP (**E**) for livers of 5-week-old WT, PTEN^ΔL^, SCAP^ΔL^, and PTEN/SCAP^ΔL^ mice. Scale bars: 100 μm. (**F**) Primary hepatocytes isolated from *Pten^fl/fl^/Scap^fl/fl^* mice were infected with Ad-Cont or Ad-Cre. The indicated proteins were assessed by WB 96 hours after infection. (**G** and **H**) At 24 hours after infection of *Pten^fl/fl^/Scap^fl/fl^* hepatocytes with Ad-Cont or Ad-Cre, culture media were changed to normal FBS media, moderately delipidated FBS (30% lipid compared with normal FBS), or completely delipidated FBS (0% lipid). At 72 hours, expression levels of indicated proteins were determined by WB analyses (**G**). At 96 hours, cell death was assessed using the Cell Death Detection ELISA Kit (*n* = 4 per group) (**H**). (**I**–**K**) Effects of GRP78 overexpression in PTEN/SCAP^ΔL^ mouse livers. We intravenously injected 4-week-old PTEN/SCAP^ΔL^ mice with 1 × 10^9^ PFU of Ad-Cont or Ad-GRP78. One week later, liver injury was assessed by H&E staining. (**I**) and serum ALT (Ad-Cont, *n* = 3; Ad-GRP78, *n* = 6) (**J**). Scale bars: 100 μm. Expression levels of GRP78 and CHOP in the liver were determined by WB analyses (**K**). Statistical data were assessed using 1-way ANOVA with Tukey’s multiple comparisons test (**C**) or Student’s *t* test (**H** and **J**). Data are presented as mean ± SEM. *P < 0.05.

To elucidate the mechanisms of liver injury in PTEN/SCAP^ΔL^ mice, we conducted RNA-Seq analyses using liver tissues obtained from 5-week-old WT, PTEN^ΔL^, SCAP^ΔL^, and PTEN/SCAP^ΔL^ mice (*n* = 3 per group) and observed the activation of the UPR pathway as well as the inflammatory signaling pathways (IL-6/JAK/STAT signaling and IFN-γ response) in PTEN/SCAP^ΔL^ mice (Figure 4A). UPR is activated in response to ER stress and plays important roles in NASH and HCC development (6). In particular, ER stress–responsive genes involved in cell death, including *Ddit3* (CHOP), *Ppp1r15a* (GADD34), *Trib3* (TRB3), *Bcl2l11* (Bim), and *Tnfrsf10b* (DR5), were upregulated in PTEN/SCAP^ΔL^ mice, which was confirmed by real-time PCR (Figure 4, B and C). WB analyses revealed increased expression of UPR signaling molecules, CHOP, phosphorylated-eIF2α (p-eIF2α), cleaved ATF6, and phosphorylated JNK (p-JNK) in PTEN/SCAP^ΔL^ mice (Figure 4D). IHC analyses showed that CHOP was broadly and strongly expressed in both the cytoplasm and nuclei in hepatocytes of PTEN/SCAP^ΔL^ mice (Figure 4E). Electron microscopy revealed ER lumen dilation in hepatocytes of PTEN/SCAP^ΔL^ mouse livers, indicating altered ER homeostasis (Supplemental Figure 7, A and B). To eliminate the influence of environmental factors such as inflammation on ER stress, we isolated primary hepatocytes from Cre-negative *Pten^fl/fl^/Scap^fl/fl^* mice and then induced gene recombination using adenovirus-expressing Cre-recombinase (Ad-Cre) (PTEN/SCAP^Δ/Δ^ hepatocytes) or control adenovirus expressing the LacZ gene (Ad-cont) (PTEN/SCAP^fl/fl^ hepatocytes). Ad-Cre efficiently induced gene recombination of *Pten* and *Scap* and increased the expression of ER stress markers, indicating that ER stress was caused by the deletion of PTEN and SCAP in a hepatocyte-autonomous manner (Figure 4F). Interestingly, the viability of PTEN/SCAP^Δ/Δ^ hepatocytes was comparable to that of PTEN/SCAP^fl/fl^ hepatocytes when cultured in medium containing normal FBS; however, under lipid-depleted conditions, PTEN/SCAP^Δ/Δ^ hepatocytes were more vulnerable to cell death accompanied by increased CHOP expression (Figure 4, G and H). These findings suggest that PTEN/SCAP^Δ/Δ^ hepatocytes depend on exogenous lipids for their survival and ER homeostasis.

Moreover, we determined whether ER stress contributes to liver injury in PTEN/SCAP^ΔL^ mice through adenoviral overexpression of ER chaperon protein GRP78, which attenuates ER stress. GRP78 overexpression significantly decreased CHOP protein levels and attenuated liver injury in PTEN/SCAP^ΔL^ mice (Figure 4, I–K). Thus, ER stress plays an important role in liver injury in PTEN/SCAP^ΔL^ mice.

### Altered phospholipid composition in PTEN/SCAP^ΔL^ mice.

**Figure 5:**
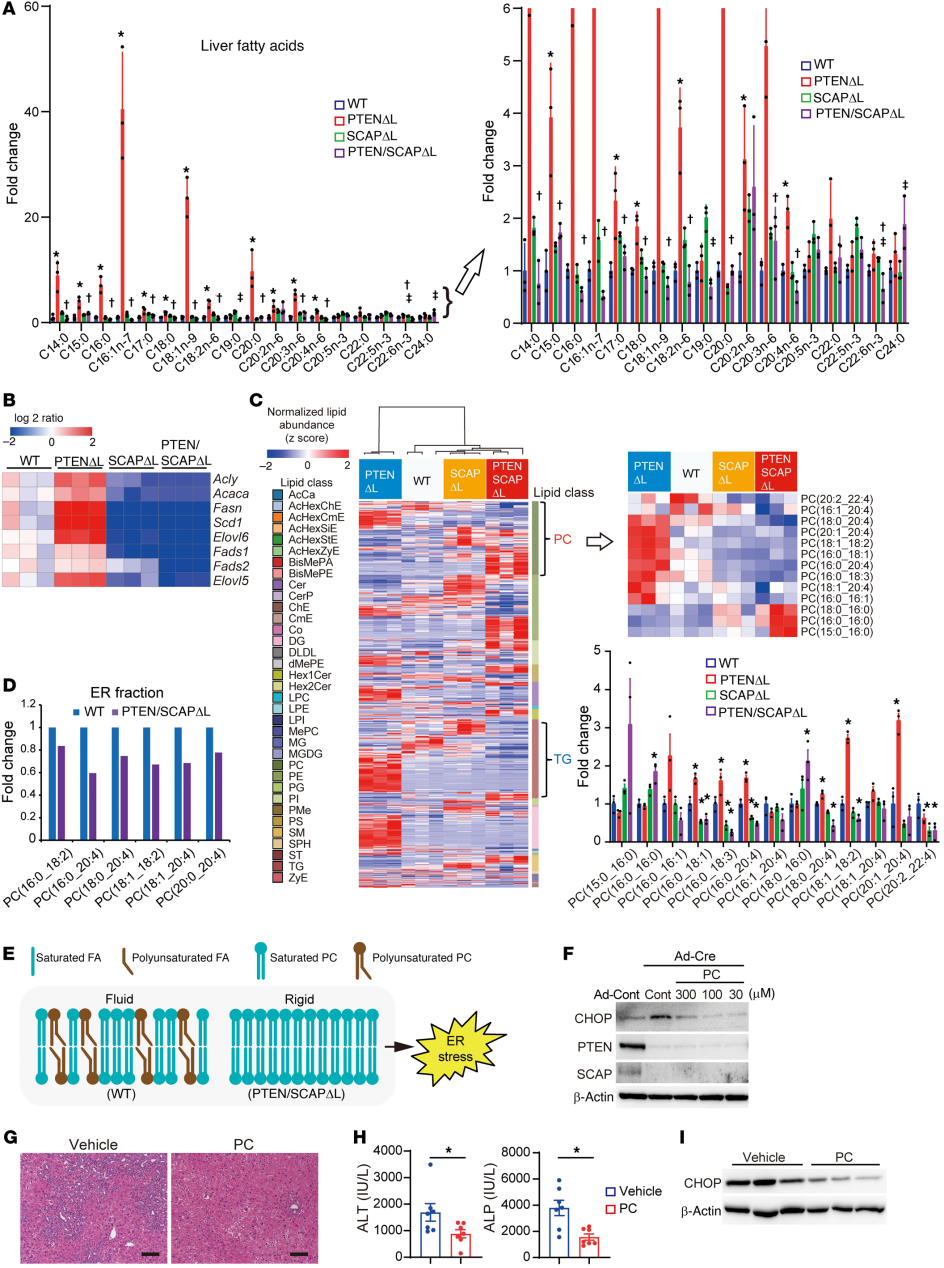
Comprehensive lipidomic analyses identified altered phospholipid composition in PTEN/SCAP^ΔL^ mice. (**A**) Liver FA contents of 5-week-old WT, PTEN^ΔL^, SCAP^ΔL^, and PTEN/SCAP^ΔL^ mice analyzed by GC-MS. Data are expressed as fold changes relative to the average in WT mice (*n* = 3 per group). *P < 0.05, WT versus PTEN^ΔL^; ^†^*P* < 0.05, PTEN^ΔL^ versus PTEN/SCAP^ΔL^; ^‡^*P* < 0.05, SCAP^ΔL^ versus PTEN/SCAP^ΔL^. Right panel shows enlargement of the lower range of the data. (**B**) Relative expression levels of lipogenesis genes determined by RNA-Seq. (**C**) Hierarchical clustering analyses of LC-MS results for liver tissues from 5-week-old WT, PTEN^ΔL^, SCAP^ΔL^, and PTEN/SCAP^ΔL^ mice. Bar graph shows relative amounts of PC species (*n* = 3 per group). *P < 0.05 compared with WT mice. (**D**) ER fractions extracted from 3 livers of 5-week-old WT or PTEN/SCAP^ΔL^ mice were pooled and analyzed for FA composition of PCs using LC-MS. (**E**) Model of membrane fluidity. A double bond in the unsaturated FA results in a bend in the string of carbon that gives the ER membrane a fluid character. (**F**) Primary hepatocytes isolated from *Pten^fl/fl^/Scap^fl/fl^* mice were infected with Ad-Cont or Ad-Cre, and PTEN/SCAP^Δ/Δ^ hepatocytes were treated with ER-targeting liposomes enriched for PC(16:0_20:4) and PC(18:0_20:4) or saline (control). At 96 hours, the indicated proteins were assessed by WB. (**G**–**I**) We orally administered a PC cocktail or vehicle to 4-week-old PTEN/SCAP^ΔL^ mice once daily; liver injury was assessed 1 week later. (**G**) H&E images of livers. Scale bars: 100 μm. (**H**) ALT and ALP serum levels (means ± SEM, *n* = 7 per group). *P < 0.05. (**I**) WB analysis of CHOP protein in the liver. Statistical data were assessed using 1-way ANOVA with Tukey’s multiple comparison test (**A**), Dunnett’s test (**C**), and Student’s *t* test (**H**).

Next, in view of the original function of SREBP, we focused on lipid metabolism. To clarify the lipid profile of the liver, we performed gas chromatography–mass spectrometry (GC-MS) analyses using liver tissues obtained from 5-week-old WT, PTEN^ΔL^, SCAP^ΔL^, and PTEN/SCAP^ΔL^ mice. As expected, FAs of various carbon chain lengths, including polyunsaturated FAs (PUFAs), were significantly increased in PTEN^ΔL^ mouse livers, but significantly decreased by additional deletion of SCAP (Figure 5A). Consistent with these results, RNA-Seq analyses revealed that the expression of enzymes for the catalysis of de novo lipogenesis and PUFA synthesis, including *Fads1*, *Fads2*, and *Elovl5*, was significantly decreased by SCAP deletion (Figure 5B); these results were confirmed by real-time PCR (Supplemental Figure 8A).

To further understand the entire lipid profile of the liver, we conducted comprehensive lipidomic analyses of liver tissues from 5-week-old WT, PTEN^ΔL^, SCAP^ΔL^, and PTEN/SCAP^ΔL^ mice using liquid chromatography–MS (LC-MS). Hierarchical clustering analyses identified changes in various lipid species via deletion of PTEN and/or SCAP (Figure 5C). For example, triacylglycerol species were expectedly increased in PTEN^ΔL^ mouse livers, but decreased by SCAP deletion. Among various lipid species, we focused on phospholipids, particularly phosphatidylcholine (PC), whose composition in the liver was greatly altered by SCAP deletion (Figure 5C). PC is the most abundant membrane phospholipid and is composed of choline, phosphate, and 2 FA chains. Saturated FAs are preferably incorporated into the sn-1 position and unsaturated FAs into the sn-2 position, and the proper fatty acyl composition of PCs is required for ER membrane biophysical characteristics (28, 29). Notably, PTEN/SCAP^ΔL^ mouse livers showed a decrease in PCs containing long-chain unsaturated FAs (LCUFAs) and PUFAs such as C18:1, C18:2, and C20:4 (Figure 5C), the loss of which causes ER membrane fluidity impairment and ER stress (28, 29). PCs containing LCUFAs and PUFAs were also decreased in lipids extracted from the ER fraction of PTEN/SCAP^ΔL^ mouse livers (Figure 5D). These findings led us to hypothesize that impaired SREBP-mediated lipogenesis may disturb the FA composition of PCs, leading to ER stress and liver injury in PTEN/SCAP^ΔL^ mice (Figure 5E). To test this hypothesis, we delivered PCs containing PUFAs using ER-targeting liposomes enriched for PC(16:0_20:4) and PC(18:0_20:4) to primary hepatocytes (30). As shown in Figure 5F, treatment with PCs containing PUFAs suppressed CHOP expression in PTEN/SCAP^Δ/Δ^ hepatocytes, whereas supplementation with C20:4 alone did not decrease CHOP expression (Supplemental Figure 8B). Moreover, oral supplementation of a PC cocktail to PTEN/SCAP^ΔL^ mice significantly improved liver injury, which was accompanied by reduced CHOP expression in vivo (Figure 5, G–I).

To further examine the vulnerability of the ER membrane through lipogenesis inhibition, we challenged *Scap^fl/fl^* and SCAP^ΔL^ mice with carbon tetrachloride (CCl4), which causes ER membrane damage, leading to acute liver injury (31, 32). As expected, SCAP^ΔL^ mice exhibited more severe liver damage, with massive hemorrhagic necrosis (Supplemental Figure 8C).

### SREBP dysfunction–mediated downregulation of lysophosphatidylcholine acyltransferase 3 is associated with ER stress.

**Figure 6:**
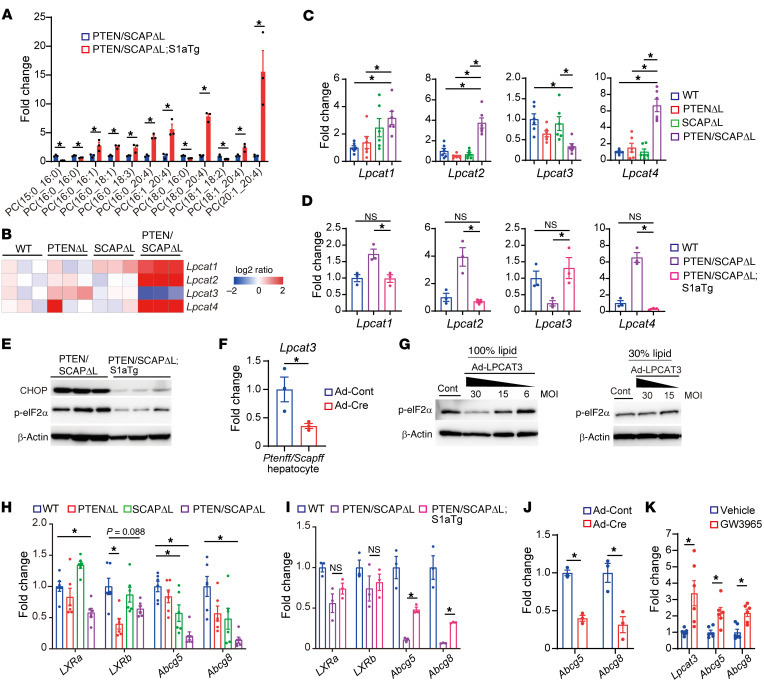
SREBP dysfunction–mediated downregulation of LPCAT3 is associated with ER stress. (**A**) FA composition of PCs in 5-week-old PTEN/SCAP^ΔL^ and PTEN/SCAP^ΔL^;S1aTg mouse livers (*n* = 3 per group). (**B** and **C**) Relative expression levels of LPCAT family members determined by RNA-Seq (**B**) and real-time PCR (**C**) in 5-week-old mice of each genotype (*n* = 6 per group). (**D**) Relative expression levels of LPCAT family members in 5-week-old mice of each genotype by real-time PCR (*n* = 3 per group). (**E**) Expression levels of indicated proteins determined by WB. (**F**) *Pten^fl/fl^/Scap^fl/fl^* hepatocytes were infected with Ad-Cont or Ad-Cre. At 96 hours, expression levels of *Lpcat3* were determined by real-time PCR (*n* = 3 per group). (**G**) Primary hepatocytes from PTEN/SCAP^ΔL^ mice were infected with Ad-Cont (MOI = 30) or Ad-LPCAT3 at indicated MOI. At 96 hours, p-eIF2α expression levels were determined by WB. Experiments were performed in medium containing normal FBS (left) or moderately delipidated FBS (30% lipid) (right). (**H**) Relative expression levels of indicated genes in livers from 5-week-old mice of each genotype by real-time PCR (*n* = 6 per group). (**I**) Expression levels of indicated genes by real-time PCR (*n* = 3 per group). (**J**) Relative expression levels of LXR target genes in hepatocytes indicated in **F** by real-time PCR (*n* = 3 per group). (**K**) PTEN/SCAP^ΔL^ hepatocytes were treated with 5 μM GW3965 or vehicle. At 48 hours, expression levels of indicated genes were analyzed by real-time PCR (*n* = 6 per group). Statistical data were assessed using Student’s *t* test (**A**, **F**, **J** and **K**) and 1-way ANOVA with Tukey’s multiple comparisons test (**C**, **D**, **H**, and **I**). Data are presented as mean ± SEM. *P < 0.05.

Next, we analyzed the effects of restoring SREBP function on the FA composition of PCs using PTEN/SCAP^ΔL^;S1aTg mouse livers. GC-MS revealed that both saturated and unsaturated FAs with various carbon chain lengths were increased following the restoration of SREBP function (Supplemental Figure 9A). However, LC-MS revealed that PCs containing LCUFAs and PUFAs, particularly C20:4, were preferentially increased in PTEN/SCAP^ΔL^;S1aTg mice, whereas PCs composed of only saturated FAs, which were increased in PTEN/SCAP^ΔL^ mice, were decreased in PTEN/SCAP^ΔL^;S1aTg mice (Figure 6A). Based on these findings, we hypothesized that not only FA biosynthesis, but also FA incorporation into PCs, might be disorganized in PTEN/SCAP^ΔL^ mice. Lysophosphatidylcholine acyltransferase (LPCAT) family members catalyze the incorporation of FAs at the sn-2 position, thereby playing critical roles in modulating the FA composition of PCs (33, 34). We examined the expression of LPCAT family members in mouse livers using RNA-Seq data and real-time PCR. In PTEN/SCAP^ΔL^ mouse livers, the expression of *Lpcat1*, *Lpcat2*, and *Lpcat4* significantly increased, whereas that of *Lpcat3* significantly decreased (Figure 6, B and C). Of note, restoring SREBP function in PTEN/SCAP^ΔL^ mouse livers corrected imbalanced expression among LPCAT family members; this was accompanied by a significant reduction in UPR (Figure 6, D and E).

Among LPCAT family members, LPCAT1 preferentially incorporates saturated FAs into PCs, while LPCAT3 incorporates PUFAs; decreased expression of LPCAT3 has also been shown to enhance ER stress (29, 35, 36). Therefore, we next focused on LPCAT3. To eliminate the influence of environmental factors on hepatic LPCAT3 expression, we analyzed LPCAT3 expression in PTEN/SCAP^fl/fl^ and PTEN/SCAP^Δ/Δ^ hepatocytes, in which gene recombination had been induced in vitro, as shown in Figure 4F. LPCAT3 expression was significantly decreased in PTEN/SCAP^Δ/Δ^ hepatocytes compared with PTEN/SCAP^fl/fl^ hepatocytes (Figure 6F). Next, we isolated primary hepatocytes from PTEN/SCAP^ΔL^ mice and analyzed the effects of LPCAT3 overexpression on ER stress. The protein levels of p-eIF2α decreased depending on the expression levels of LPCAT3 (Figure 6G and Supplemental Figure 9B), suggesting that LPCAT3 downregulation is involved in ER stress in PTEN/SCAP^ΔL^ mouse hepatocytes. In contrast, LPCAT3 overexpression did not attenuate UPR under lipid-depleted conditions in PTEN/SCAP^ΔL^ mouse hepatocytes (Figure 6G), indicating that sufficient lipids and the ability to incorporate FA into PCs are both required to maintain ER homeostasis.

LPCAT3 expression is regulated by the liver X receptor (LXR), a member of the nuclear receptor family of transcription factors (29). The mRNA levels of LXRα (*Nr1h3*) and LXRβ (*Nr1h2*) were moderately decreased, and those of its target genes, *Abcg5* and *Abcg8*, were markedly decreased in PTEN/SCAP^ΔL^ mice compared with those of WT mice (Figure 6H). LXR target gene expression levels were also mildly decreased in SCAP^ΔL^ mice compared with WT mice, whereas LXRα expression was increased. The restoration of SREBP function to PTEN/SCAP^ΔL^ mouse livers partially but significantly upregulated LXR target genes without increasing LXR expression (Figure 6I). These findings suggest that the deletion of PTEN and SCAP synergistically downregulated the expression of LXR target genes and that *SCAP* deletion may suppress LXR transcriptional activity. The expression levels of LXR target genes were also significantly decreased in PTEN/SCAP^Δ/Δ^ hepatocytes compared with PTEN/SCAP^fl/fl^ hepatocytes in vitro (Figure 6J), indicating that the downregulation of LXR activity is a cell-autonomous effect. The LXR agonist GW3965 significantly upregulated the expression of LPCAT3 as well as LXR target genes in PTEN/SCAP^ΔL^ mouse–derived primary hepatocytes (Figure 6K). Furthermore, GW3965 improved liver injury in PTEN/SCAP^ΔL^ mice (Supplemental Figure 9C).

Sterol intermediates from the cholesterol biosynthetic pathway are endogenous ligands for LXR (37, 38). To elucidate the downregulation of LXR activity in PTEN/SCAP^ΔL^ mouse livers, we performed LC–MS/MS to quantify sterol intermediates in WT and PTEN/SCAP^ΔL^ mouse livers. Although the expression of enzymes involved in cholesterol biosynthesis such as *Sqle* and *Dhcr7* was significantly decreased in PTEN/SCAP^ΔL^ mice (Figure 1G), sterol intermediates that have been shown to activate LXR were instead increased in PTEN/SCAP^ΔL^ mice (Supplemental Figure 9D), probably due to decreased biliary cholesterol secretion caused by severe liver injury. In fact, the restoration of SREBP function to PTEN/SCAP^ΔL^ mice decreased most sterol intermediates in the liver despite increased expression of cholesterol biosynthesis enzymes (Figure 3C and Supplemental Figure 9D). Thus, endogenous LXR ligands that have not yet been identified may be involved in LXR downregulation in PTEN/SCAP^ΔL^ mice.

### Analysis of human NAFLD samples.

**Figure 7:**
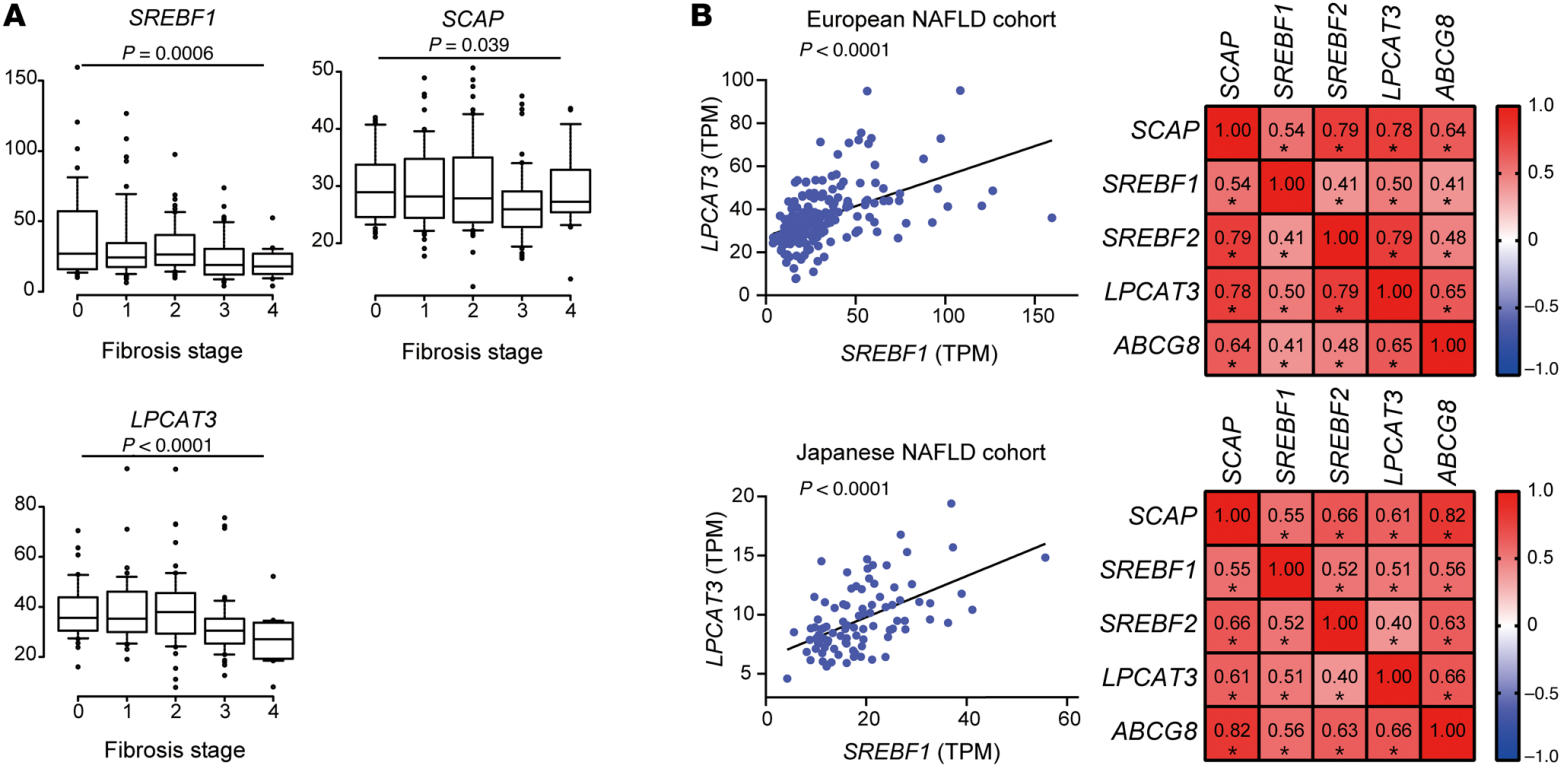
Analyses of human samples. (**A**) Hepatic expression levels of *SREBF1*, *SCAP*, and *LPCAT3* according to the fibrosis stage in European NAFLD cohort (F0, *n* = 38; F1, *n* = 47; F2, *n* = 53; F3, *n* = 54; F4, *n* = 14). Box plot shows the mean (horizontal line), interquartile range (box), 10th and 90th percentiles (whiskers), and outliers outside the 10th and 90th percentiles (dots). The decreasing tendencies of gene expression levels across the fibrosis stage were assessed using the Jonckheere-Terpstra trend test. (**B**) Left panels show scatterplots of the hepatic expression levels of *SREBF1* and *LPCAT3*, and right panels show Spearman’s rank-correlation matrix (upper panels, European cohort; lower panels, Japanese cohort).*P < 0.05.

Although hepatic steatosis is a hallmark of NAFLD, hepatic fat deposition is often significantly decreased in advanced NASH, referred to as “burned-out NASH.” Importantly, the SREBP-mediated lipogenesis pathway is reportedly downregulated in advanced NASH (39). To confirm this, we analyzed publicly available transcriptome data with histological findings of liver biopsies obtained from 206 European patients with NAFLD (40). Consistent with the previous report, the expression level of *SREBF1* gradually decreased with the progression of fibrosis (Figure 7A). Interestingly, the expression levels of *SCAP* and *LPCAT3* showed similar trends (Figure 7A). In addition, as in the mouse model, there were significant positive correlations among *SCAP*, *SREBF1*, *SREBF2*, *LPCAT3*, and *ABCG8* (Figure 7B). Moreover, we performed RNA-Seq analysis using liver biopsy samples obtained from 94 Japanese patients with NAFLD at our institute. Similar relationships among *SCAP*, *SREBF1*, *SREBF2*, *LPCAT3*, and *ABCG8* were observed in this cohort (Figure 7B), suggesting that there exists the pathway via the SCAP/SREBP/LXR/LPCAT3 axis in human NASH also and that its downregulation may be involved in the pathogenesis of advanced burned-out NASH.

### Deletion of SCAP cooperates with mTOR activation to trigger liver injury.

**Figure 8:**
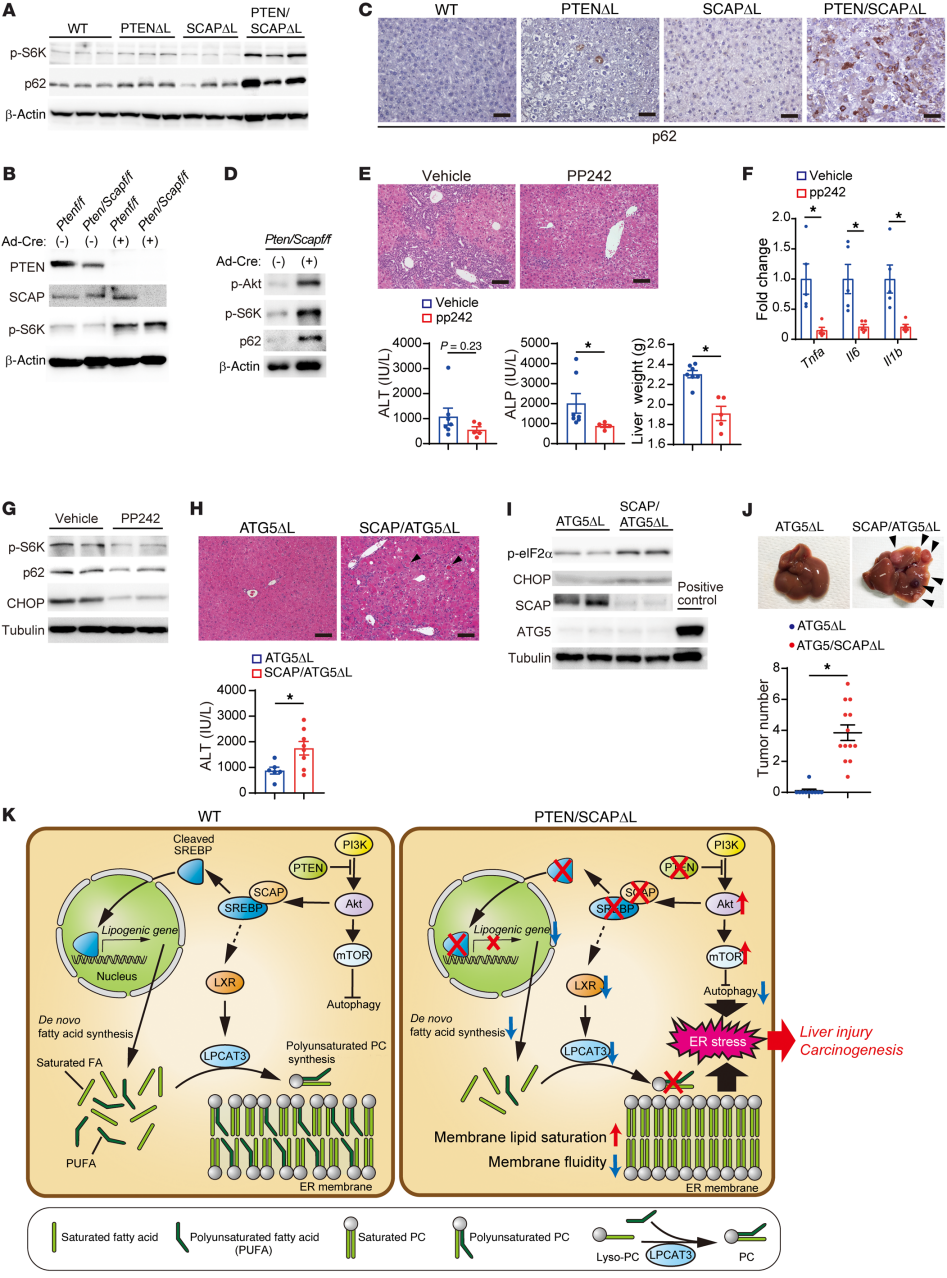
Deletion of *SCAP* cooperated with mTOR activation to trigger liver injury. (**A**) WB analyses of the indicated proteins for livers from 5-week-old WT, PTEN^ΔL^, SCAP^ΔL^, and PTEN/SCAP^ΔL^ mice. (**B**) *Pten^fl/fl^* and *Pten^fl/fl^/Scap^fl/fl^* primary hepatocytes were infected with Ad-Cont or Ad-Cre. At 96 hours, the indicated proteins were assessed by WB. (**C**) IHC images of p62 for livers from mice indicated in **A**. Scale bars: 100 μm. (**D**) *Pten^fl/fl^/Scap^fl/fl^* primary hepatocytes were infected with Ad-Cont or Ad-Cre. At 96 hours, the indicated proteins were assessed by WB. (**E**–**G**) Effects of mTOR inhibitor (PP242) administration on PTEN/SCAP^ΔL^ mouse livers. We orally administered PP242 (60 mg/kg) or vehicle control to 4-week-old PTEN/SCAP^ΔL^ mice once daily and assessed liver injury 1 week later. (**E**) H&E staining images, ALT and ALP serum levels, and liver weight are shown (vehicle, *n* = 7; PP242, *n* = 5). Scale bars: 100 μm (**F**) Relative expression levels of inflammatory cytokines analyzed by real-time PCR (vehicle, *n* = 5; PP242, *n* = 5). (**G**) WB analyses of the indicated liver proteins. (**H**–**J**) Effects of double knockout of ATG5 and SCAP on the liver. (**H**) H&E images of livers and serum ALT in 2-month-old ATG5^ΔL^ and SCAP/ATG5^ΔL^ mice are shown (ATG5^ΔL^, *n* = 6; SCAP/ATG5^ΔL^, *n* = 8). Scale bars: 100 μm. (**I**) WB analyses of indicated proteins in livers. (**J**) Representative liver images and tumor numbers in 10-month-old ATG5^ΔL^ and SCAP/ATG5^ΔL^ mice (ATG5^ΔL^, *n* = 10; SCAP/ATG5^ΔL^, *n* = 13). Arrowheads indicate liver tumors. (**K**) Schematic representation of our proposed model. All statistical data were assessed using Student’s *t* test (**E**, **F**, **H**, and **J**). Data are presented as mean ± SEM. *P < 0.05.

Although PCs containing LCUFAs were also decreased in SCAP^ΔL^ mouse livers (Figure 5C), SCAP^ΔL^ mice did not exhibit spontaneous liver injury, suggesting that an additional factor induced by PTEN deletion is required for liver injury. PTEN deletion activates the Akt/mTOR pathway, which induces autophagy inhibition and increases of protein synthesis, both of which enhance ER stress (41–43). We examined the expression levels of phosphorylated ribosomal S6 kinase (p-S6K), a substrate of mTOR, and found that p-S6K levels were slightly increased in PTEN^ΔL^ mice and markedly increased in PTEN/SCAP^ΔL^ mice (Figure 8A). To examine the effects of PTEN single knockout and PTEN/SCAP double knockout on S6K phosphorylation in vitro, we isolated primary hepatocytes from Cre-negative *Pten^fl/fl^* and *Pten^fl/fl^/Scap^fl/fl^* mice and introduced gene recombination using Cre-expressing adenovirus. The 2 cell types showed similar increases in S6K phosphorylation, suggesting that phosphorylation of S6K in PTEN/SCAP^ΔL^ mice is enhanced by environmental factors such as inflammatory cytokines and/or growth factors in vivo (Figure 8B). Indeed, the p-S6K levels in PTEN-knockout hepatocytes (PTEN^Δ/Δ^ hepatocytes) were significantly enhanced by supplementation with hepatocyte growth factor (HGF), which increased in PTEN/SCAP^ΔL^ mouse livers and reportedly enhanced S6K phosphorylation (Supplemental Figure 10, A and B, and ref. 44). In addition, p62 protein, a substrate of autophagy, was significantly increased in PTEN/SCAP^ΔL^ mice, and IHC analyses revealed increased p62 aggregates in PTEN/SCAP^ΔL^ mice, indicating autophagy inhibition (Figure 8, A and C). Increased p62 protein levels were also observed in PTEN/SCAP^Δ/Δ^ hepatocytes in vitro, which was more pronounced compared with those in PTEN^Δ/Δ^ hepatocytes (Figure 8D and Supplemental Figure 10C). Accumulation of p62 is induced by ER stress combined with impaired autophagy (45); the ER stress marker CHOP was also increased in PTEN/SCAP^Δ/Δ^ hepatocytes compared with PTEN^Δ/Δ^ hepatocytes (Supplemental Figure 10C). GRP78 overexpression in PTEN/SCAP^ΔL^ mice reduced p62 expression (Supplemental Figure 10D), suggesting that not only impaired autophagy but also ER stress is involved in the increase in the p62 protein levels in PTEN/SCAP^ΔL^ mice.

To determine whether mTOR activation is involved in liver injury in PTEN/SCAP^ΔL^ mice, we treated PTEN/SCAP^ΔL^ mice with mTOR inhibitor PP242. The expression levels of p-S6K, p62, CHOP, and inflammatory cytokines decreased, which tended to improve liver injury in PTEN/SCAP^ΔL^ mice (Figure 8, E–G). Because autophagy plays an important role in clearing damaged organelles, including the ER, we hypothesized that defective autophagy due to mTOR activation may impair the removal of damaged ER with disturbed composition of membrane PCs, resulting in ER stress and cellular damage. To test this hypothesis, we specifically inhibited autophagy in SCAP^ΔL^ mice through additional deletion of the autophagy-essential gene *Atg5* by crossing SCAP^ΔL^ mice with *Atg5^fl/fl^* mice (SCAP/ATG5^ΔL^). Although the liver-specific ATG5 single-knockout mice generated by crossing *Atg5^fl/fl^* and *Alb-Cre* mice (ATG5^ΔL^) developed spontaneous liver injury as previously reported (46), the additional deletion of SCAP in ATG5^ΔL^ mice significantly aggravated liver injury, with enhanced expression of ER stress markers (Figure 8, H and I). Furthermore, liver tumor development was significantly enhanced in SCAP/ATG5^ΔL^ mice compared with ATG5^ΔL^ mice at 10 months of age (Figure 8J). Together, these results demonstrate that the deletion of SCAP cooperates with mTOR activation to trigger liver injury, which can be partially explained by autophagy inhibition (Figure 8K).

## Discussion

We observed the unexpected exacerbation of liver injury, fibrosis, and carcinogenesis following inhibition of SREBP-mediated lipogenesis in a murine NASH model. These findings are critical to developing NASH treatment strategies because they indicate that excessively broad and strong inhibition of the lipogenic pathway may be counterproductive in NASH therapy. Importantly, hepatic fat deposition is often decreased in advanced stage NASH accompanied by SREBP downregulation (39), and this finding was confirmed also in the present study. Although the significance of downregulation of the SREBP pathway in advanced NASH has been unknown, our present study raises the possibility that downregulation of the SREBP pathway may be a disease-promoting factor in advanced stage NASH. In fact, less steatosis in patients with NASH-related cirrhosis is associated with higher risk for HCC development and mortality, even among patients with the same Child-Turcotte-Pugh score (47). Thus, both excessive and insufficient SREBP activation may result in NASH disease progression, and appropriate SREBP activity may be essential for maintaining liver homeostasis.

We demonstrated that changes in the FA composition of phospholipids due to SCAP/SREBP pathway inhibition are involved in ER stress and liver injury in PTEN/SCAP^ΔL^ mice. The appropriate FA composition of PCs plays an important role in maintaining the structure and function of biological membranes, and the PUFA content of PCs is a particularly important factor influencing membrane fluidity (28, 29). High membrane fluidity is required for vesicular trafficking, and impaired vesicular transport causes accumulation of misfolded proteins in the ER, eventually leading to ER stress (48). The restoration of SREBP function in PTEN/SCAP^ΔL^ mice broadly upregulated de novo FA and PUFA synthesis, whereas only PCs containing PUFAs were increased and PCs containing only saturated FAs were instead decreased in PTEN/SCAP^ΔL^;S1aTg mice. Therefore, we considered that abnormal PC composition in PTEN/SCAP^ΔL^ mouse livers was caused by both impaired PUFA synthesis and disorganized FA incorporation into PCs. The expression of LPCAT family members that catalyze FA incorporation at the sn-2 position in PCs was dramatically altered in PTEN/SCAP^ΔL^ mice and rescued by the restoration of SREBP function. Among LPCAT family members, we focused on the downregulation of LPCAT3, the key enzyme for PUFA incorporation into PCs, and found that the introduction of LPCAT3 into PTEN/SCAP^ΔL^ mouse–derived hepatocytes reduced UPR. A previous study reported that LPCAT3 knockdown enhanced ER stress cooperatively with SCD1 knockdown (35), further supporting a synergistic effect of the impairment of FA biosynthesis and FA incorporation into PCs on ER stress. We also showed that the activity of LXR, a key transcription factor regulating LPACT3 expression, was downregulated in PTEN/SCAP^ΔL^ mouse livers and that an LXR agonist restored LPCAT3 expression in PTEN/SCAP^ΔL^ mouse–derived hepatocytes. Thus, LXR-mediated LPCAT3 expression was impaired in PTEN/SCAP^ΔL^ mouse livers, which may partially explain the observed decrease in PCs containing PUFAs.

The concentrations of sterol intermediates, which are endogenous ligands for LXR, were high in PTEN/SCAP^ΔL^ mice and reduced by restoration of SREBP function. We considered that this phenomenon was due to decreased biliary cholesterol secretion caused by severe liver injury in PTEN/SCAP^ΔL^ mice. However, Rong et al. reported that liver-specific SREBP2-knockout mice show reduced LXR activity in the liver despite increased amounts of sterol intermediates, especially desmosterol (11). Although the liver phenotype of liver-specific SREBP2-knockout mice was not described in Rong’s study, liver injury was not apparent. Therefore, downregulation of LXR activity despite increased sterol intermediates may be specific to SREBP-2 dysfunction, and there might be an as-yet-unknown SREBP-2–dependent endogenous LXR ligand. In addition, activity of sterol intermediates to LXR reportedly differs among cell types. For example, desmosterol activates LXR in macrophages, but not in hepatocytes (49), possibly explaining the absence of an increase in LXR activity in PTEN/SCAP^ΔL^ mice despite increased sterol intermediates.

Several recent studies have demonstrated altered phospholipid metabolism in patients with NASH. Puri et al. (50) analyzed the lipid composition of liver biopsy specimens and found that total PC amounts were decreased in patients with NAFLD and NASH compared with healthy subjects and that PCs containing C20:4 were particularly decreased in NASH patients. Hall et al. (51) investigated the distribution of phospholipids in liver tissues using MS imaging and reported that PCs containing C20:4 showed a characteristic distribution around the portal vein in normal livers, which was increasingly disrupted as the disease progressed from NAFLD to NASH and cirrhosis. Furthermore, in the present study, we found that the expression levels of *LPCAT3* gradually decreased with the progression of fibrosis in NASH patients. A recent clinical study showed that PC supplementation improved liver enzymes in patients with NAFLD (52). Thus, phospholipid metabolism disturbance may be a potential therapeutic target for NASH.

We considered that the PTEN^ΔL^ mouse would be an appropriate model to analyze the role of the SREBP pathway in NASH because disease progression is assumed to be more dependent on SREBP-mediated de novo lipogenesis in this model than in HFD-induced models, in which liver steatosis predominantly depends on the influx of FAs from outside of the liver. Although the PTEN^ΔL^ mouse model does not replicate the full spectrum of human NASH (e.g., obesity and insulin resistance), the impaired autophagy and increased protein synthesis that underlie liver injury pathogenesis in PTEN/SCAP^ΔL^ mice have also been reported in human NASH (53, 54). Moreover, enhanced hepatocarcinogenesis through SCAP deletion was observed in another NASH model, the CDAHFD model. Therefore, we consider the results obtained in this study to be transferable to human NASH, to some extent. However, although SREBP activity decreases with the progression of liver steatosis, inflammation, and fibrosis in clinical settings, SREBP activity was inhibited by nature in our mouse model. Further work, such as an inducible SCAP-knockout study, is needed to address this issue.

In conclusion, the strong inhibition of SCAP/SREBP-mediated lipogenesis unexpectedly exacerbated liver injury, fibrosis, and carcinogenesis in murine NASH via the disturbance of phospholipid metabolism. These findings have important implications for the development of NASH treatment strategies.

## Methods

### Animal experiments.

*Alb-Cre*, *Scap^fl/fl^*, *Rosa26*–*Lox*–*Stop*–*Lox*–*tdTomato*, and *S1aTg* mice were purchased from the Jackson Laboratory (18, 27). *Pten^fl/fl^* and *Atg5^fl/fl^* mice were provided by Tak W. Mak (University of Toronto, Toronto, Canada) and Noboru Mizushima (University of Tokyo, Tokyo, Japan), respectively (55, 56). *Pten^fl/fl^* and *Atg5^fl/fl^* mice were of the C57BL/6 genetic background, and *Scap^fl/fl^* and *S1aTg* mice were backcrossed into the C57BL/6 strain at least 10 times. Only male mice were used for tumorigenesis and metabolomics analyses.

CDAHFD (A06071302) was purchased from Research Diets. CCl4 was diluted in corn oil to a 1:4 ratio and injected intraperitoneally into mice (2 mL/kg). A PC cocktail (Merck) was dissolved in corn oil containing 10% ethanol and administered once daily by oral gavage at 100 mg/kg. PP242 (Wako) was dissolved in a solution of 20% DMSO, 40% polyethylene glycol-400, and 40% PBS and administered to mice once daily by oral gavage at 60 mg/kg. GW3965 (Sigma-Aldrich) was dissolved in corn oil containing 10% DMSO and administered at 40 mg/kg to mice, once daily by oral gavage. AAV-control, AAV-nSREBP-1c, or AAV-nSREBP-2 was constructed by Vector Biolabs, and Ad-Cont, Ad-Cre, and Ad-LPCAT3 were purchased from that company. Ad-GRP78 was provided by Randal J. Kaufman (Sanford Burnham Prebys Medical Discovery Institute, La Jolla, California, USA) (6).

### Histology.

Mouse livers were fixed in 10% neutral buffered formalin or 4% paraformaldehyde, embedded in paraffin, and sectioned. For IHC, fixed and paraffin-embedded liver sections were deparaffinized and incubated in Target Retrieval Solution (Dako) buffer at 95°C for 35 minutes for antigen retrieval and then incubated overnight at 4°C with the primary antibodies. Biotinylated secondary antibodies (Pharmingen) were added and incubated for 20 minutes at room temperature. Streptavidin-horseradish peroxidase (Pharmingen) was added, and after 30 minutes, the sections were developed with 3,3′-diaminobenzidine (DAB) substrate (Vector Laboratories) and counterstained with hematoxylin. For immunofluorescence, slides were incubated with primary antibodies, followed by secondary antibodies labeled with Alexa Fluor 488 or 555 (Invitrogen). Oil red O staining was performed according to the manufacturer’s protocol (Wako). Sirius red staining was performed by Septsapie. The stained area was quantified using ImageJ software (NIH). TUNEL staining was performed using the Apoalert DNA Fragmentation Assay Kit (Clontech). The number of TUNEL^+^ cells was counted manually in at least 4 fields (magnification, ×100) per slide.

For electron microscopy, livers were fixed with 2.5% glutaraldehyde and 1% osmic acid, embedded in Epon 812, and cut into ultrathin sections. The sections were doubly stained with uranyl acetate and lead acetate and examined under an electron microscope (JEM 1011, JEOL).

### Immunoblotting, RNA extraction, and real-time PCR.

Immunoblotting was performed as previously described (57). RNA was extracted from liver tissues using ISOGEN with a spin column (Nippon Gene). First-strand cDNA was synthesized using the iScript cDNA Synthesis Kit (Bio-Rad). The relative amount of each mRNA was quantified via real-time PCR and normalized against *Gapdh* mRNA expression. Primer sequences are listed in Supplemental Table 1.

### Antibodies for immunostaining and immunoblotting.

Detailed antibody information is given in Supplemental Table 2.

### ER fractionation and nuclear protein extraction from mouse liver.

The ER fraction of the mouse liver was extracted using the Endoplasmic Reticulum Enrichment Kit (Novus Biologicals) according to the manufacturer’s protocol. ER samples obtained from 3 livers of each genotype were pooled, and the final pellet was suspended in 100 μL PBS for lipidomic analyses. Cytoplasmic and nuclear protein fractions were extracted from mouse liver using Subcellular Protein Fractionation Kit for Tissues (Thermo Scientific).

### Cell culture.

Mouse primary hepatocytes were isolated following a 2-step collagenase digestion protocol and then cultured in William’s E medium with 10% FBS on collagen-coated plates. For lipid starvation, cells were rinsed with serum-free medium and then placed in medium containing 10% delipidated FBS (Gemini Bio). We prepared 30% lipid medium by mixing normal FBS and delipidated FBS at a ratio of 3:7. Cell death was evaluated using the Cell Death Detection ELISA Kit (Merck). GW3965 (Sigma-Aldrich) was dissolved in DMSO and added to medium at 5 μM. C20:4 (Tokyo Chemical Industry Co.) was dissolved in ethanol. For experiments using adenoviral vectors, mouse primary hepatocytes were infected with each adenoviral vector at 24 hours after isolation. After overnight incubation, the virus-containing medium was removed and replaced with fresh medium. Cells were infected with Ad-Cre at a multiplicity of infection (MOI) of 60 for Cre-loxP–dependent gene recombination. PC(16:0_20:4) and PC(18:0_20:4) were purchased from Avanti Polar Lipids, and ER-targeting liposome (PC[16:0_20:4]:PC[18:0_20:4]:dioleoyl-phosphatidylethanolamine:dipalmitoyl-phosphatidylserine at a molar ratio of 1:1:2:1) was generated by Beacle Inc.

### Analyses of human samples.

Liver biopsy samples were collected from 94 patients with biopsy-proven NAFLD at the University of Tokyo Hospital from May 2016 to December 2020 (49 men, 45 women; median age 54 yr; 25 to 75th percentile, 44–68 yr). The recruitment criteria of liver biopsy was as follows: a transient liver elastographic value (measured by Fibroscan) above 7.0 kPa, persistent elevation of serum aspartate aminotransferase and ALT for at least 6 months, a fatty liver diagnosed ultrasonically by an increase in hepatorenal contrast, a history of alcohol consumption of less than 30 g/d for men and less than 20 g/d for women, seronegativity for hepatitis B virus surface antigen and hepatitis C virus antibody, and the absence of autoimmune hepatitis, primary biliary cholangitis, primary sclerosing cholangitis, Budd-Chiari syndrome, Wilson disease, and drug-induced liver injury. All samples were collected in the morning of the day of liver biopsy after an overnight fast. Three mm of liver biopsy sample was reserved at –70°C until analysis. For RNA-Seq analysis, extracted RNA samples were processed with the TruSeq Stranded Total RNA LT Sample Prep Kit (Gold, Illumina) and sequenced on a NovaSeq6000 system (Illumina). Raw sequencing reads were aligned to a human reference genome (GRCh38) using STAR (58). Gene-level count and TPM tables were produced using RSEM (59). Sequence data were deposited in the NCBI’s Gene Expression Omnibus database (GEO GSE174478).

The gene-level read count data of the European cohort was obtained from GEO GSE135251 (40). Gene-level TPM values were calculated with nonoverlapping exon length using GenomicFeatures package (60).

### RNA-Seq of mouse samples.

In RNA-Seq analysis of mouse liver tumor samples, the sequencing library was prepared using the 2000 mg TruSeq RNA Library Prep Kit (version 2, Illumina) according to the manufacturer’s protocol. Raw sequencing reads were aligned to a mouse reference genome (mm39) using STAR, and gene-level count tables were produced using RSEM (59). The resulting gene counts were used as input for differential expression analysis using DESeq2 (61). Statistical significance of protein-coding genes was determined by an FDR-corrected *q* of less than 0.05 and fold change of greater than |1.5|. Sequence data were deposited in GEO (GSE174173). Also, in RNA-Seq analysis of mouse liver samples obtained at 5 weeks of age, the sequencing library was prepared using the 2000 mg TruSeq RNA Library Prep Kit (version 2, Illumina). Raw sequencing reads were aligned to a mouse reference genome (mm10) using the STAR algorithm (58), and gene expression levels were normalized using the DESeq2 method (61). Molecular pathway dysregulation in liver tissues was determined by gene set enrichment analyses, surveying molecular pathway gene sets obtained from the HALLMARK database (62). Sequence data were deposited in GEO (GSE169104).

### Exome sequence analyses.

DNA was isolated from liver tumors and spleens using the QIAamp DNA Mini Kit (QIAGEN). DNA samples from spleens were used as germline controls. PTEN^ΔL^ samples consisted of 7 tumors and 1 spleen, and PTEN/SCAP^ΔL^ samples consisted of 8 tumors and 1 spleen. DNA was sequenced using the Sureselect Human All Exon V6 platform (Veritas Genetics). Mutations were analyzed using a bioinformatics approach, as follows. The Trim Galore script (Babraham Bioinformatics) was used to discard short reads and reads with insufficient base quality. The trimmed reads were aligned to the reference genome (GRCm38) using the Burrows-Wheeler aligner (BWA), which requires separately generated index files. Several postprocessing steps were required to prepare the files for single-nucleotide variant (SNV), loss of heterozygosity, and copy number variation calling. The CleanSam tool (Samtools) was used to obtain information on soft-clipped reads, which were only partly aligned to the reference genome. Next, these files were sorted using the Samtools algorithm (Samtools). The Picard Readgroups tool (Samtools) was used to mark reads that were sequenced together, followed by duplicate reads. Base recalibration was conducted in the final step of post-processing. Somatic point mutations and indels were called simultaneously and stored as.vcf files by the Mutect2 program (Genome Analysis Toolkit [GATK]). We removed probable technical or germline artifacts using the FilterMutectCalls function within the GATK package and filtered all indels greater than 10 bp using the SelectVariants function. We filtered for mutant allele frequency of 10% or more, 10× or greater coverage at particular positions in tumor and normal samples, and at least 3 supporting reads for the mutation in the tumor sample. To further reduce the rate of false-positive calls, we compared SNVs and indels to known polymorphisms listed by the Wellcome Sanger Institute. Sequence data were deposited in the DNA Data Bank of Japan (DDBJ) database (DRA011741).

### Metabolome analyses.

For hepatic TG and cholesterol content, lipids were extracted with chloroform/methanol (2:1) and analyzed by Skylight Biotech. GC-MS and LC-MS analyses were conducted at the Kazusa DNA Research Institute as previously described (63, 64). The resulting data were analyzed using LipidSearch software (Kazusa DNA Research Institute). To quantify cholesterol metabolites, total lipids were extracted from mouse livers using a butanol/methanol method with internal standards (65). Focused lipidomic analyses for cholesterol metabolites were performed using a triple-quadrupole mass spectrometer (LCMS-8060, Shimadzu) equipped with a Nexera Ultra-Performance LC System (Shimadzu). MS/MS analyses were conducted in positive-ion and negative-ion modes, and the cholesterol metabolites were identified and quantified via multiple-reaction monitoring at the Kazusa DNA Research Institute as previously reported (66, 67).

### Statistics.

Statistical significance was defined as *P* < 0.05. The number of tumors larger than 2 mm was counted for comparative analyses of tumor development. All in vitro experiments were performed at least 3 times independently. Data analyses were performed using Graphpad Prism software (version 9.0) or R statistical software (www.r-project.org).

### Study approval.

All animal experiments were approved by the Ethics Committee for Animal Experimentation of the University of Tokyo and the Institute for Adult Diseases, Asahi Life Foundation, and were conducted in accordance with the NIH *Guidelines for the Care and Use of Laboratory Animals* (National Academies Press, 2011). Analysis of human samples was approved by the University of Tokyo Medical Research Center Ethics Committee (approval numbers 1302 and 3955) and was performed in accordance with the ethical guidelines of the Declaration of Helsinki. All patients provided written, informed consent.

## Author contributions

S Kawamura performed the experiments, analyzed the data, and wrote the paper. YM analyzed transcriptomic and metabolomic data and wrote the paper. S Kurosaki, M Tange, Y Hayata, Y Hayakawa, NS, MH, M Tsuboi, TK, and HK helped with some experiments and data interpretation and edited the manuscript. NF and Y Hoshida helped with metabolomics and transcriptomics data analysis. TN, MS, YK, and RT provided clinical samples. AU and TI provided critical materials. AE, Y Hirata, MU, KT, and KK edited the manuscript, with important intellectual input. HN conceived and designed the study and wrote the paper. MF edited the manuscript, with important intellectual input.

## Supplementary Material

Supplemental data

## Version 1 04/05/2022

In-Press Preview

## Version 2 06/01/2022

Electronic publication
