# Supplementary material for: Inhibiting SCAP/SREBP exacerbates liver injury and carcinogenesis in murine nonalcoholic steatohepatitis
Source: J Clin Invest. 2022 Jun 1;132(11):e151895. doi: 10.1172/JCI151895 (PMC9151706; doi:10.1172/JCI151895)
Supplement: Supplemental data [file jci-132-151895-s127.pdf]

Supplemental Figure 1

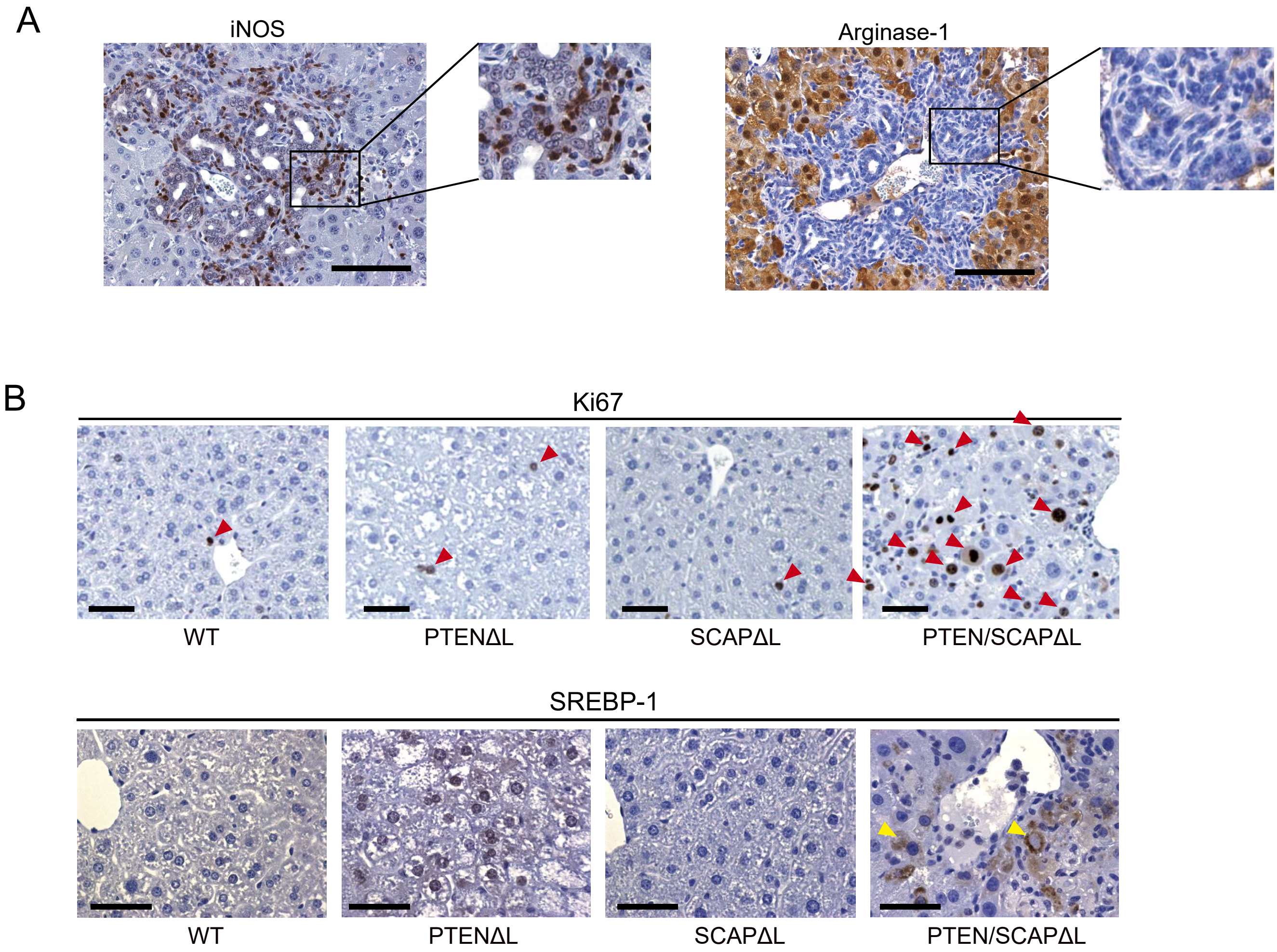

Supplemental Figure 1. iNOS$^+$ macrophages infiltrate PTEN/SCAP$^{\Delta L}$ mouse liver. (A) IHC images of iNOS and arginase-1 in 5-week-old PTEN/SCAP$^{\Delta L}$ mouse liver (scale bar, 100 μm). (B) Enlarged high-magnification images of Figure 1D (scale bar, 100 μm). Red arrowheads, Ki67-expressing hepatocytes; yellow arrowheads, hepatocytes expressing SREBP-1 in the cytoplasm but not the nucleus.

Supplemental Figure 2

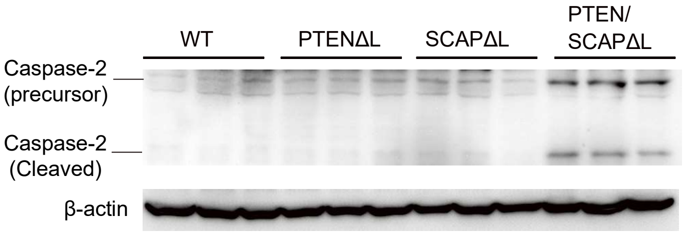

Supplemental Figure 2. Caspase-2 activation in PTEN/SCAP$^{\Delta L}$ mice. Western blotting analysis of caspase-2 activation in livers from 5-week-old WT, PTEN$^{\Delta L}$, SCAP$^{\Delta L}$, and PTEN/SCAP$^{\Delta L}$ mice.

Supplemental Figure 3

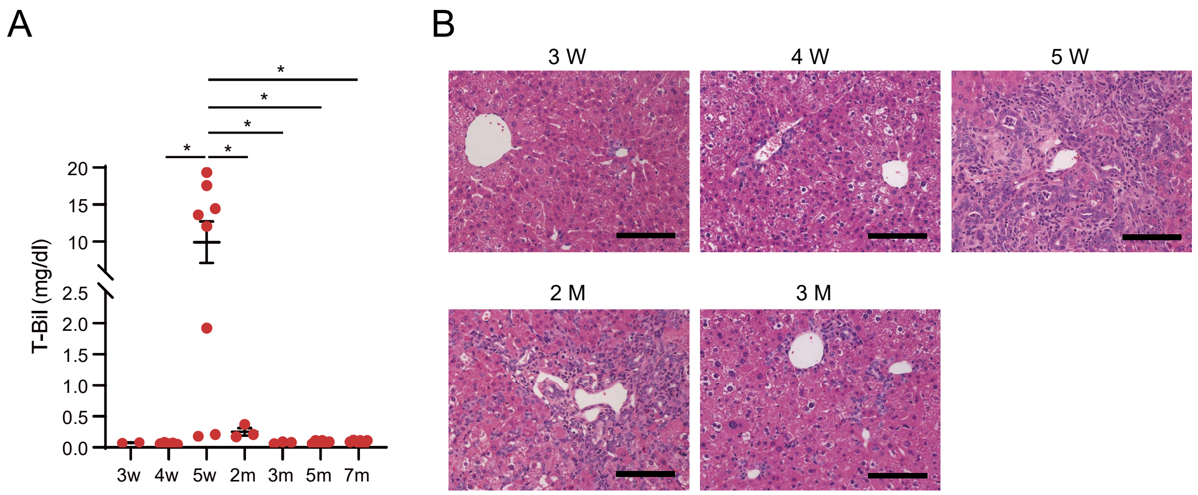

Supplemental Figure 3. Time course analysis of liver injury in PTEN/SCAP$^{\Delta L}$ mice. (A) Time course of serum total bilirubin levels in PTEN/SCAP$^{\Delta L}$ mice (sample size per age: 3 weeks, n = 2; 4 weeks, n = 5; 5 weeks, n = 8; 2 months, n = 3; 3 months, n = 3; 5 months, n = 6; 7 months, n = 6). Statistical data were assessed using 1-way ANOVA with Tukey's multiple comparison test; *$p < 0.05$. (B) H&E stained images of livers from PTEN/SCAP$^{\Delta L}$ mice at indicated time points (scale bar, 100 μm).

Supplemental Figure 4

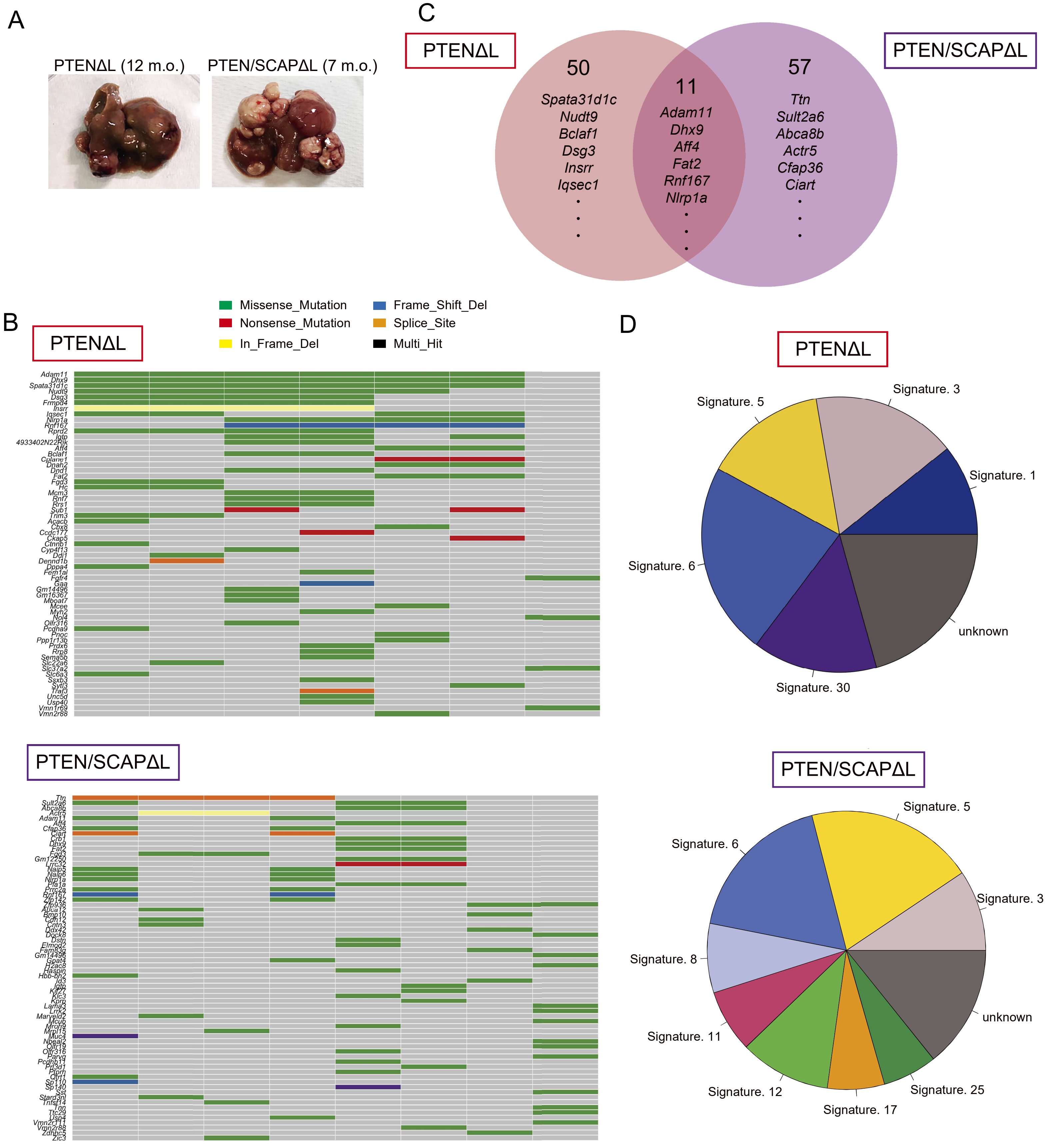

Supplemental Figure 4. Whole-exome sequencing of liver tumors from PTEN$^{\Delta L}$ and PTEN/SCAP$^{\Delta L}$ mice. (A–D) Whole-exome sequencing of liver tumor samples obtained from 12-month-old PTEN$^{\Delta L}$ mice (n = 7) and 7-month-old PTEN/SCAP$^{\Delta L}$ mice (n = 8). (A) Representative macroscopic images of livers from 12-month-old PTEN$^{\Delta L}$ and 7-month-old PTEN/SCAP$^{\Delta L}$ mice. (B) Heat maps showing non-synonymous somatic mutation profiles. (C) Venn diagram showing shared and distinct mutations between PTEN$^{\Delta L}$ and PTEN/SCAP$^{\Delta L}$ tumors. The top six mutated genes in each category are shown. (D) Pie charts showing mutational signatures according to COSMIC nomenclature.

Supplemental Figure 5

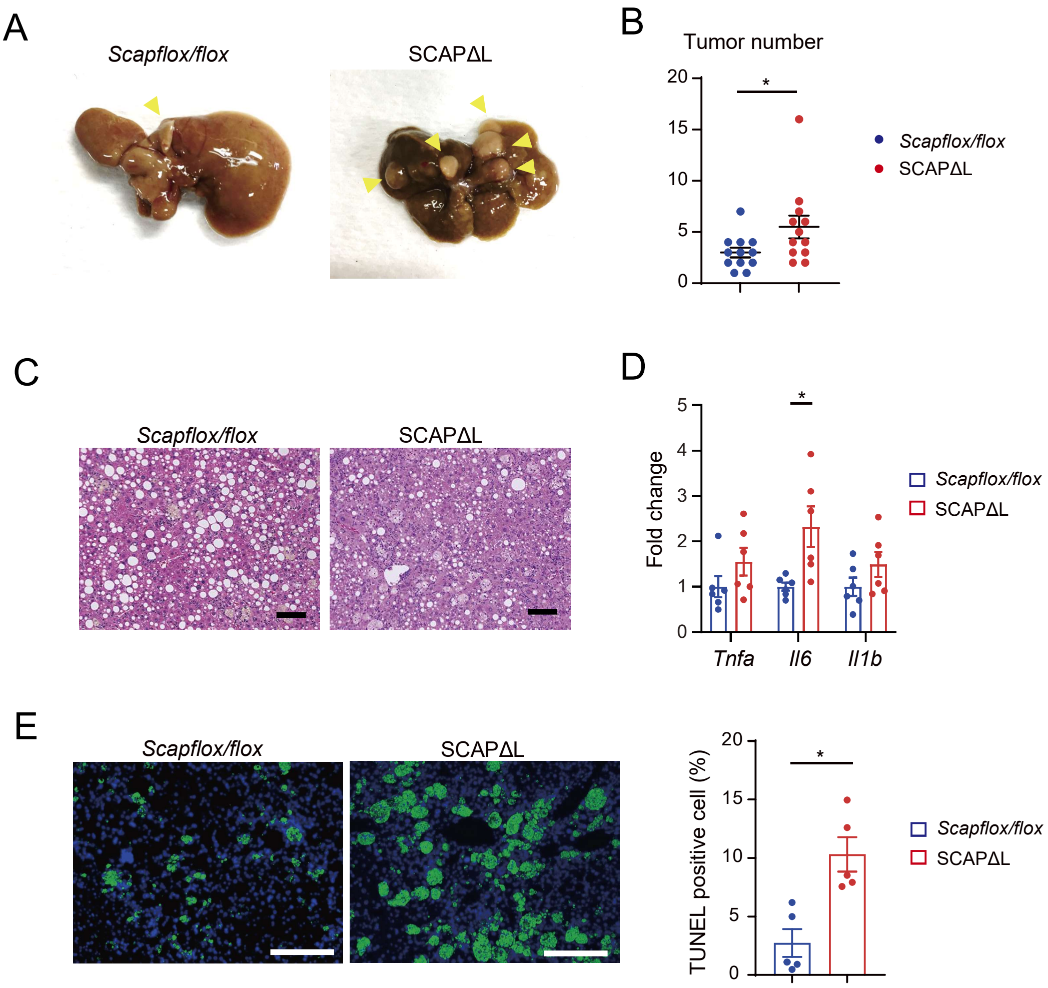

Supplemental Figure 5. Effect of SCAP deletion on a CDAHFD-induced NASH–HCC model. (A–C) Representative macroscopic images (scale bar, 100 μm) (A), tumor numbers (B), and H&E stained images of livers from *Scap*$^{flox/flox}$ and SCAP$^{\Delta L}$ mice placed on CDAHFD for 32 weeks (n = 12 per group). (D) Relative expression levels of inflammatory cytokines in non-tumor liver tissues of *Scap*$^{flox/flox}$ and SCAP$^{\Delta L}$ mice placed on CDAHFD for 32 weeks were determined by real-time PCR (n = 6 per group). (E) TUNEL-stained images of the livers of CDAHFD-fed *Scap*$^{flox/flox}$ and SCAP$^{\Delta L}$ mice (scale bar, 200 μm). Bar graph shows the percentage of TUNEL-positive hepatocytes (n = 5 per group). All statistical data were assessed using Student's *t*-test. Data are presented as means ± SEM; *$p < 0.05$.

Supplemental Figure 6

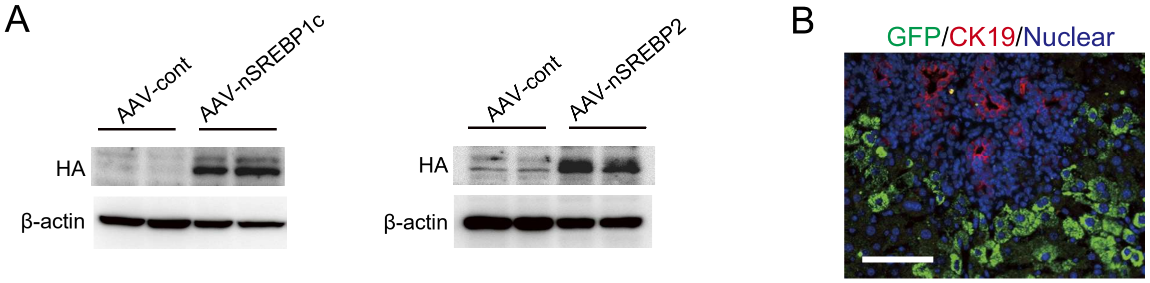

Supplemental Figure 6. AAV-mediated delivery of nSREBP-1c or nSREBP-2 into PTEN/SCAP$^{\Delta L}$ mouse livers. (A) Introduction of nSREBP-1c or nSREBP-2 was confirmed by Western blotting analyses with anti-HA-tag antibody. (B) We intravenously injected 1.5×10^{11} genome copies of AAV-eGFP (AAV-control) into 4-week-old PTEN/SCAP$^{\Delta L}$ mice; 1 week later, eGFP expression in the liver was examined by immunostaining. Double immunofluorescence staining with eGFP (green) and CK19 (red) is shown (scale bar, 100 μm).

Supplemental Figure 7

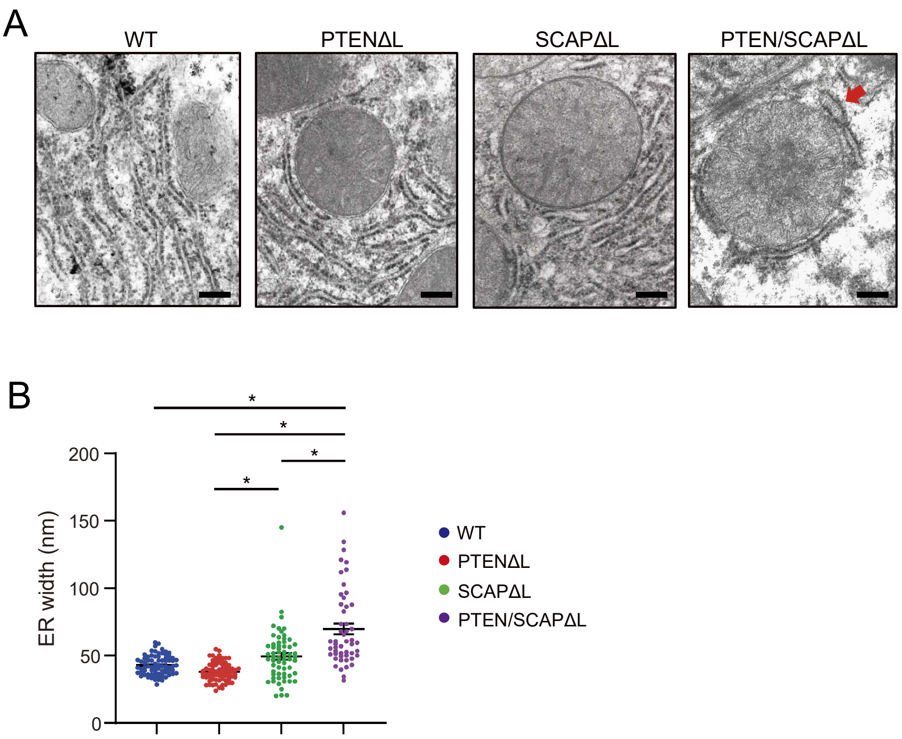

Supplemental Figure 7. Liver electron microscopic images. (A) Electron microscopic images of livers from 5-week-old WT, PTEN$^{\Delta L}$, SCAP$^{\Delta L}$, and PTEN/SCAP$^{\Delta L}$ mice are shown (scale bar = 200 nm). Red arrow indicates dilated endoplasmic reticulum (ER). (B) ER width in each mouse genotype. 1-way ANOVA with Tukey's multiple comparison test was used for statistical analysis; *$p < 0.05$.

Supplemental Figure 8

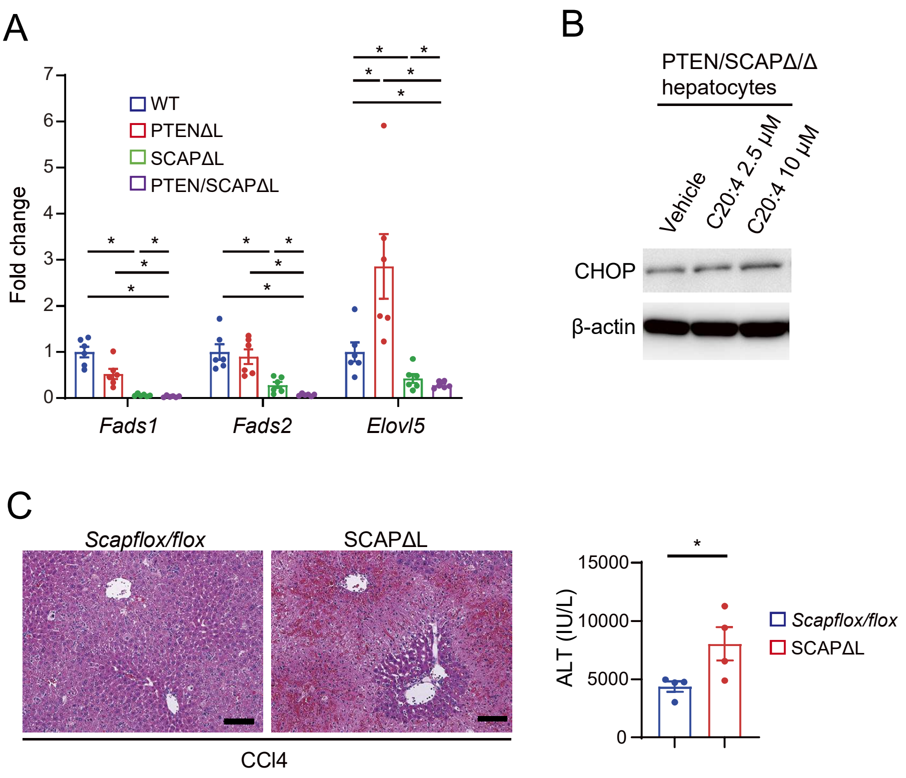

Supplemental Figure 8. Inhibition of PUFA synthesis and vulnerability of the ER membrane in PTEN/SCAP$^{\Delta L}$ mice. (A) Relative expression levels of lipogenesis genes involved in PUFA synthesis were analyzed by real-time PCR in livers from 5-week-old WT, PTEN$^{\Delta L}$, SCAP$^{\Delta L}$, and PTEN/SCAP$^{\Delta L}$ mice (n = 6 per group). (B) Primary hepatocytes isolated from *Pten$^{flox/flox}$/Scap$^{flox/flox}$* mice were infected with Ad-Cre. At 24 h after infection, PTEN/SCAP$^{\Delta/\Delta}$ hepatocytes were treated with the indicated concentrations of C20:4 (10 μM was the maximum dose that did not affect cell viability). At 96 h, CHOP expression was assessed by WB. (C) Effect of hepatic SCAP deletion on CCl4-induced acute liver injury. *Scap$^{flox/flox}$* and SCAP$^{\Delta L}$ mice were injected intraperitoneally with 2 mg/kg CCl4; at 24 h, liver injury was assessed. H&E staining images of livers (scale bar, 100 μm) and ALT serum levels are shown (n = 4 per group). Statistical data in A was assessed using 1-way ANOVA with Tukey's multiple comparison test, and in C using Student's *t*-test. Data are presented as means ± SEM; *$p < 0.05$.

Supplemental Figure 9

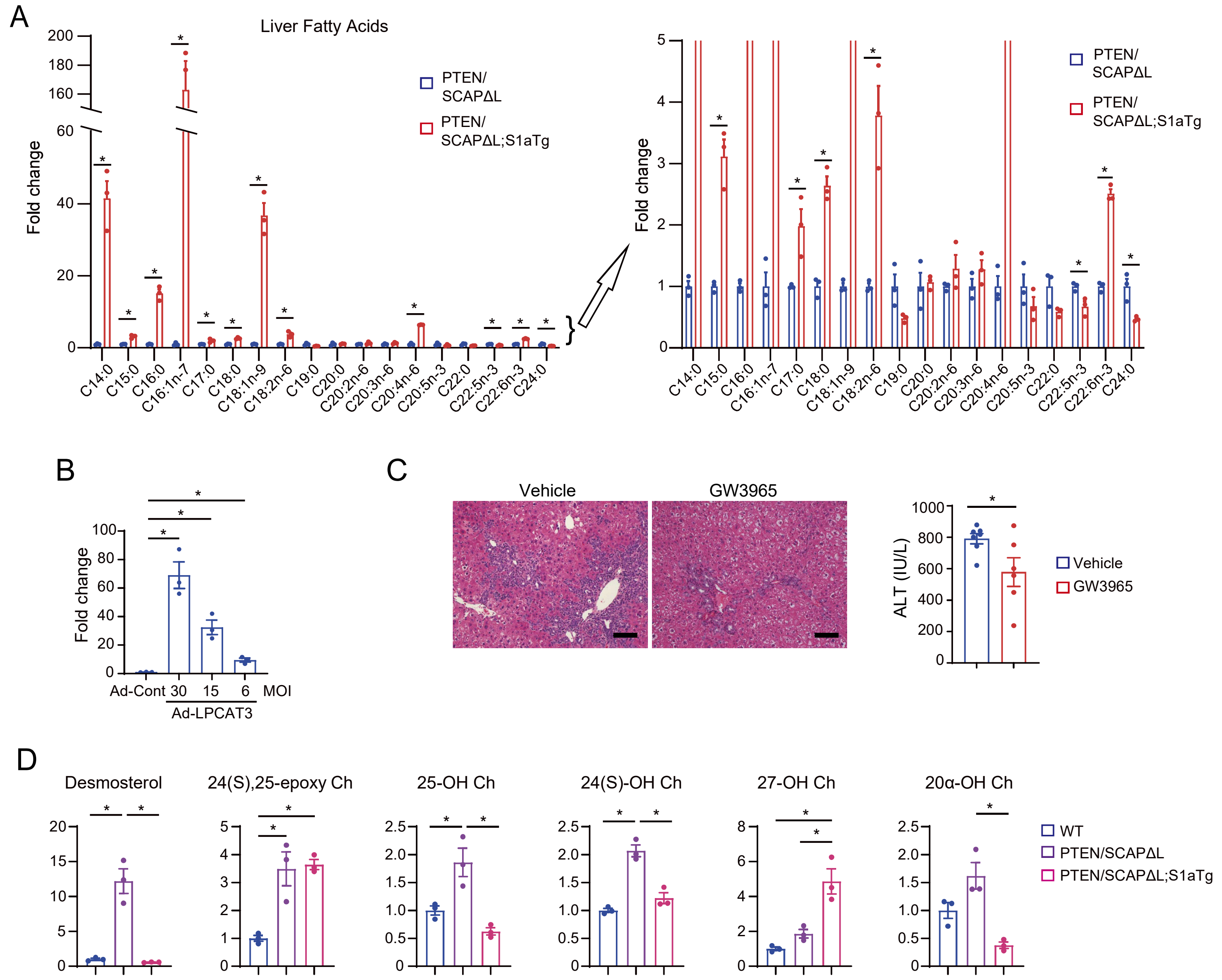

Supplemental Figure 9. Effect of SREBP restoration into PTEN/SCAP$^{\Delta L}$ mice on hepatic lipid profile. (A) Liver FA content of 5-week-old PTEN/SCAP$^{\Delta L}$ and PTEN/SCAP$^{\Delta L}$;S1aTg mice was analyzed by GC–MS; data are expressed as fold changes relative to the average in PTEN/SCAP$^{\Delta L}$ mouse livers (n = 3 per group). Right panel shows magnification of the lower range of the data. (B) Primary hepatocytes isolated from PTEN/SCAP$^{\Delta L}$ mice were infected with Ad-Cont or Ad-LPCAT3 at the indicated MOI. Ad-Cont was infected at MOI = 30. At 96 h, the expression level of LPCAT3 was analyzed by real-time PCR (n = 3 per group) (C) Effect of LXR agonist (GW3965) administration on PTEN/SCAP$^{\Delta L}$ mouse livers. We orally administered GW3965 (40 mg/kg) or vehicle control to 4-week-old PTEN/SCAP$^{\Delta L}$ mice once daily and assessed liver injury 1 week later. H&E-stained images (scale bar, 100 µm) and serum ALT levels are shown (vehicle, n = 7; GW3965, n = 6). (D) Hepatic sterol and oxysterol content of 5-week-old WT, PTEN/SCAP$^{\Delta L}$ and PTEN/SCAP$^{\Delta L}$;S1aTg mice were analyzed by mass spectrometry. Data are expressed as fold changes relative to the average in WT mouse livers (n = 3 per group). Statistical data were assessed in A and C using Student's *t*-test, in B using Dunnett's test, and in D using 1-way ANOVA with Tukey's multiple comparison test. Data are presented as means ± SEM; $*p < 0.05$.

Supplemental Figure 10

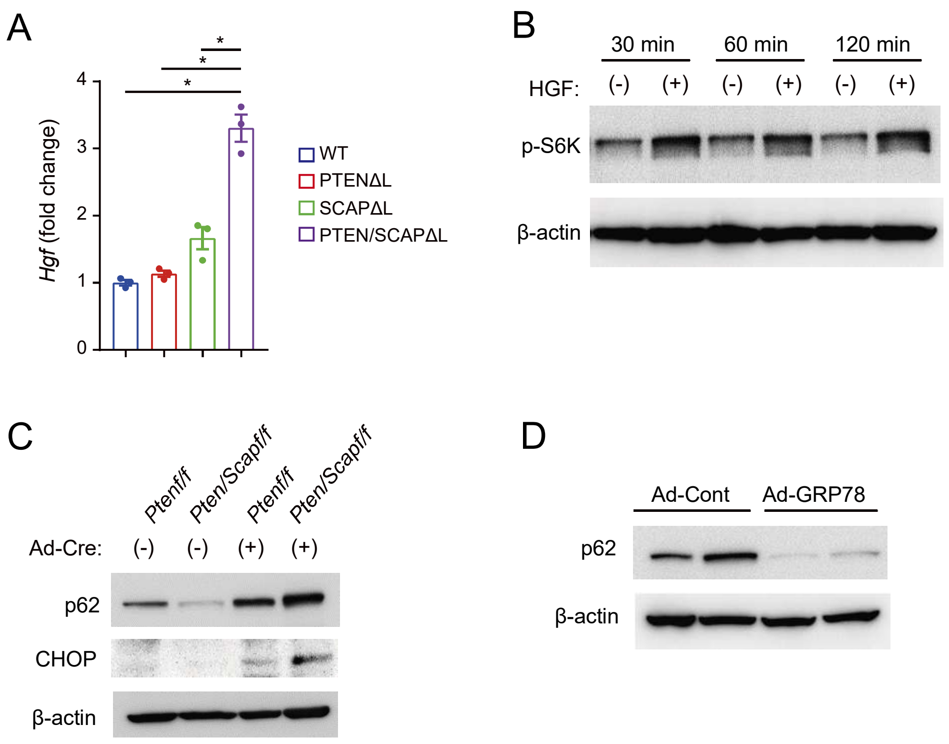

Supplemental Figure 10. The mechanisms of enhanced expression of phosphorylated S6K and p62 in PTEN/SCAP$^{\Delta L}$ mice. (A) Relative expression levels of HGF determined by RNA-seq in 5-week-old mice of each genotype (n = 3 per group). Data were assessed using 1-way ANOVA with Tukey's multiple comparison test. Data are presented as means ± SEM; **p* < 0.05. (B) Primary hepatocytes isolated from *Pten*$^{flox/flox}$ mice were infected with Ad-Cre. At 72 h after infection, PTEN$^{\Delta/\Delta}$ hepatocytes were treated with HGF (100 ng/mL) or control medium. The expression levels of p-S6K protein were assessed by WB at the indicated time points. (C) *Pten*$^{flox/flox}$ and *Pten*$^{flox/flox}$/*Scap*$^{flox/flox}$ primary hepatocytes were infected with Ad-Cont or Ad-Cre. At 96 h, the levels of the indicated proteins were assessed by WB. (D) 4-week-old PTEN/SCAP$^{\Delta L}$ mice were injected with 1 × 10^9 pfu of Ad-Cont or Ad-GRP78. 1 week later, p62 expression was assessed by WB.

Supplementary Table 1. List of real-time PCR primers

Gene	Sequences of primers (5'→3')	
Abcg5	Forward	TGGATCCAACACCTCTATGCTAAA
	Reverse	GGCAGGTTTTCTCGATGAACTG
Abcg8	Forward	TGCCCACCTTCCACATGTC
	Reverse	ATGAAGCCGGCAGTAAGGTAGA
Acaca	Forward	GAAGCCACAGTGAAATCTCG
	Reverse	GATGGTTTGGCCTTTCACAT
Bcl2l11	Forward	GAGATACGGATTGCACAGGA
	Reverse	ATTTGAGGGTGGTCTTCAGC
Col1a1	Forward	TAGGCCATTGTGTATGCAGC
	Reverse	ACATGTTCAGCTTTGTGGACC
Ddit3	Forward	TTCTGCTTTCAGGTGTGGTG
	Reverse	CATGTTGAAGATGAGCGGGT
Dhcr7	Forward	GCTCTTCGCTCCATTCATTGT
	Reverse	AAGCCAGGAATAAAGCAGCAC
Elovl5	Forward	ATGGACACCTTTTTCTTCATCCTT
	Reverse	ATGGTAGCGTGGTGGTAGACATG
Fads1	Forward	CCAGCTTTGAACCCACCAA
	Reverse	CATGAGGCCCATTCGCTCTA
Fads2	Forward	TCAAAACCAACCACCTGTTCTTC
	Reverse	GATGAACCAGGCAAGGCTTTC
Fasn	Forward	GTTGGCCCAGAACTCCTGTA
	Reverse	GTCGTCTGCCTCCAGAGC
Gapdh	Forward	TTGATGGCAACAATCTCCAC
	Reverse	CGTCCCGTAGACAAAATGGT
Hmgcr	Forward	CACAATAACTTCCCAGGGGT
	Reverse	GGCCTCCATTGAGATCCG
Il1b	Forward	GGTCAAAGGTTTGGAAGCAG
	Reverse	TGTGAAATGCCACCTTTTGA
Il6	Forward	ACCAGAGGAAATTTTCAATAGGC
	Reverse	TGATGCACTTGCAGAAAACA
Lpcat1	Forward	GGCTCCTGTTCGCTGCTTT
	Reverse	TTCCTCCATAAGGCCAGGGG

Lpcat2	Forward	GTCCAGCAGACTACGATCAGT
	Reverse	GCAGCAAAATTATTCCAACCAGT
Lpcat3	Forward	TGGGCCGCACCATCAC
	Reverse	AGTTGCCGGTGGCAGTGTA
Lpcat4	Forward	CTCATCCGATACCCCAACAGT
	Reverse	GGGAGGAACTCTACATCCACG
Nr1h3	Forward	GCAGAGCAAACTCAGCATCA
	Reverse	GGACTTCAGTTACAACCGGG
Nr1h2	Forward	GCCTGGGAATGGTTCTCCTC
	Reverse	AGATGACCACGATGTAGGCAG
Ppp1r15a	Forward	GGAGATAGAAGTTGTGGGCG
	Reverse	TTTTGGCAACCAGAACCG
Pten	Forward	CATAACCCACCACAGCTAG
	Reverse	GCAGACCACAAACTGAGG
Scap	Forward	AGAATTCCACAGGTCCCGTT
	Reverse	CTGCGCATCCTATCCAATTC
Scd1	Forward	CAGCCGAGCCTTGTAAGTTC
	Reverse	GCTCTACACCTGCCTCTTCG
Sqle	Forward	AGTTCGCTGCCTTCTCGGATA
	Reverse	GCTCCTGTTAATGTCGTTTCTGA
Tgfb1	Forward	CAACCCAGGTCCTTCCTAAA
	Reverse	GGAGAGCCCTGGATACCAAC
Tnfa	Forward	AGGGTCTGGGCCATAGAACT
	Reverse	CCACCACGCTCTTCTGTCTAC
Tnfrsf10b	Forward	TGACGGGGAAGAGGAACTGA
	Reverse	GGCTTTGACCATTTGGATTTGA
Trib3	Forward	TAGGCAGCCGGGCATAAG
	Reverse	CTTTTGGAACGAGAGCAAGG

Supplementary Table2. List of antibodies

Antibody	Host	Dilution	Cat. no.	Supplier
Arginase	Rabbit	1:400 (IHC)	93668	Cell Signaling
ATF6	Rabbit	1:1000 (WB)	65880	Cell Signaling
ATG5	Rabbit	1:1000 (WB)	12994	Cell Signaling
Caspase-2	Rat	1:1000 (WB)	ALX-804-356	Enzo Life Sciences
CD45	Rat	1:100 (IHC)	14-0451-82	eBioscience
CHOP	Rabbit	1:5000 (WB)	5554	Cell Signaling
		1:100 (IHC)		
CK19	Rat	1:100 (IHC)	MABT913	Millipore
		1:100 (IF)		
Cleaved caspase-3	Rabbit	1:100 (IHC)	9661	Cell Signaling
F4/80	Rat	1:100 (IHC)	MCA4976A	Biorad
GRP78	Rabbit	1:1000 (WB)	13968	Santa Cruz
HA	Rabbit	1:1000 (WB)	3724	Cell Signaling
HDAC1	Rabbit	1:1000 (WB)	7872	Santa Cruz
HSP90	Rabbit	1:5000 (WB)	4877	Cell Signaling
iNOS	Rabbit	1:50 (IHC)	15323	Abcam
Ki67	Rabbit	1:100 (IHC)	16667	Abcam
p62	Rabbit	1:1000 (WB)	23214	Cell Signaling
		1:100 (IHC)		
p-AKT	Rabbit	1:2000 (WB)	4060	Cell Signaling
p-eIF2α	Rabbit	1:1000 (WB)	3398	Cell Signaling
p-JNK	Rabbit	1:2000 (WB)	9251	Cell Signaling
p-S6K	Rabbit	1:1000 (WB)	97596	Cell Signaling
PTEN	Rabbit	1:5000 (WB)	9559	Cell Signaling
SCAP	Goat	1:1600 (WB)	9675	Santa Cruz
SREBP1	Rabbit	1:1000 (WB)	MABS1987	Millipore
		1:100 (IHC)		
RFP	Rabbit	1:200 (IF)	600-401-379	Rockland
Tubulin	Mouse	1:5000 (WB)	9026	Sigma-Aldrich
β-actin	Mouse	1:5000 (WB)	AC74	Sigma-Aldrich
γH2AX	Rabbit	1:100 (IHC)	9718	Cell Signaling
